# Regio-regular alternating diketopyrrolopyrrole-based D_1_–A–D_2_–A terpolymers for the enhanced performance of polymer solar cells†

**DOI:** 10.1039/c9ra08858j

**Published:** 2019-12-18

**Authors:** Myeongjae Lee, Taehyo Kim, Hoai Van T. Nguyen, Hye Won Cho, Kyung-Koo Lee, Jong-Ho Choi, BongSoo Kim, Jin Young Kim

**Affiliations:** Department of Chemistry, Korea University 145 Anam-ro Seongbuk-gu Seoul 02841 Republic of Korea; School of Energy and Chemical Engineering, Ulsan National Institute of Science and Technology (UNIST) 50 UNIST-gil Ulsan 44919 Republic of Korea jykim@unist.ac.kr +82-52-217-2909 +82-52-217-2911; Department of Chemistry, Kunsan National University 558 Daehak-ro Gunsan-si Jeollabuk-do 54150 Republic of Korea; Department of Chemistry, Ulsan National Institute of Science and Technology (UNIST) 50 UNIST-gil Ulsan 44919 Republic of Korea bongsoo@unist.ac.kr +82-52-317-2297 +82-52-317-3197; Green Materials and Processes Group, Korea Institute of Industrial Technology Ulsan 44413 Republic of Korea

## Abstract

We designed and synthesized regio-regular alternating diketopyrrolopyrrole (DPP)-based D_1_–A–D_2_–A terpolymers (PDPPF2T2DPP-T2, PDPPF2T2DPP-TVT, and PDPPF2T2DPP-DTT) using a primary donor (D_1_) [3,3′-difluoro-2,2′-bithiophene (F2T2)] and a secondary donor (D_2_) [2,2′-bithiophene (T2), (*E*)-1,2-di(thiophen-2-yl)ethene (TVT), or dithieno[3,2-*b*:2′,3′-*d*]thiophene (DTT)]. A PDPP2DT-F2T2 D–A polymer was synthesized as well to compare optical, electronic, and photovoltaic properties. The absorption peaks of the terpolymers (PDPPF2T2DPP-T2, PDPPF2T2DPP-TVT, and PDPPF2T2DPP-DTT) were longer (*λ*_max_ = 801–810 nm) than the peak of the PDPP2DT-F2T2 polymer (*λ*_max_ = 799 nm), which is associated with the high-lying HOMO levels of the terpolymers (−5.08 to −5.13 eV) compared with the level of the PDPP2DT-F2T2 polymer (−5.38 eV). The photovoltaic properties of these DPP-based polymers were investigated under simulated AM 1.5G sunlight (100 mW cm^−2^) with a conventional structure (ITO/PEDOT:PSS/polymer:PC_71_BM/Al). The open-circuit voltages (*V*_oc_) of photovoltaic devices containing the terpolymers were slightly lower (0.68–0.70 V) than the *V*_oc_ of the device containing the PDPP2DT-F2T2 polymer (0.79 V). The short-circuit current (*J*_sc_) of the PDPPF2T2DPP-DTT device was significantly improved (14.14 mA cm^−2^) compared with that of the PDPP2DT-F2T2 device (8.29 mA cm^−2^). As a result, the power conversion efficiency (PCE) of the PDPPF2T2DPP-DTT device (6.35%) was increased by 33% compared with that of the simple D–A-type PDPP2DT-F2T2 device (4.78%). The highest *J*_sc_ and PCE values (the PDPPF2T2DPP-DTT device) were attributed to an optimal nanoscopically mixed morphology and strong interchain packing with a high face-on orientation in the blend film state. The study demonstrated that our strategy of using multiple donors in a regio-regular alternating fashion could fine-tune the optical, electronic, and morphological properties of D–A-type polymers, enhancing the performance of polymer solar cells.

## Abstract 2

We designed and synthesized regio-regular alternating diketopyrrolopyrrole (DPP)-based D_1_–A–D_2_–A terpolymers (PDPPF2T2DPP-T2, PDPPF2T2DPP-TVT, and PDPPF2T2DPP-DTT) for use in polymer solar cells.

## Graphical abstract

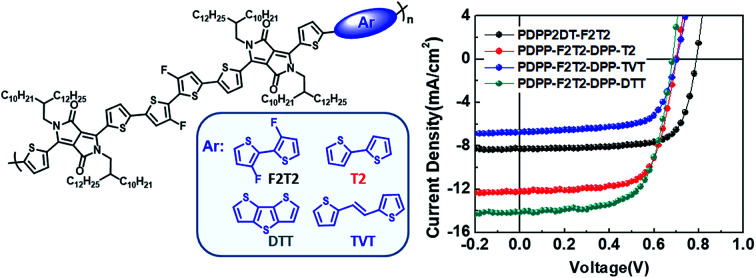

## 1. Introduction

Polymer solar cells (PSCs) have been investigated as a future energy source because of advantages such as the low-cost of source materials and production and the potential for fabricating lightweight, flexible products.^1–3^ Improving the photovoltaic properties of PSCs requires the development of appropriate donor–acceptor-type conjugated polymers consisting of an electron-donating unit (donor or D) and an electron-accepting unit (acceptor or A). D–A polymers have been synthesized and demonstrated the desired energy level, energy bandgap, and light absorption properties.^4^ In the blend film where D–A polymers and n-type molecules are combined to form a photoactive layer, they are required to exhibit high crystallinity, good carrier transport, and optimal morphology;^5^ PSC devices made using these photoactive materials would have a high open-circuit voltage (*V*_oc_), short-circuit current (*J*_sc_), fill-factor (FF), and power conversion efficiency (PCE). However, because of the synthetic difficulty in developing new donor and acceptor units, combining known D or A moiety derivatives is a common approach, which could limit the fine-tuning of the optoelectronic properties of conjugated polymers for high-performance PSCs. To overcome these limitations, the use of D_1_–A–D_2_–A terpolymers has recently emerged as an effective strategy for constructing high-performance conjugated polymers.^6,7^ D_1_–A–D_2_–A terpolymers are composed of three monomers with one acceptor unit and two different donor units in a conjugated polymer backbone. The use of the D_1_–A–D_2_–A structure allows the production of a number of conjugated copolymers by varying the D and A monomer combination in the polymerization process. Moreover, this rational polymer structural design would enhance physical properties such as energy level, energy bandgap, and light absorption properties. In particular, the regio-regular connection of D and A units seems to be preferable for high photovoltaic performance.^8,9^

2,5-Dihydropyrrolo[4,3-*c*]pyrrolo-1,4-dione (DPP) moiety, first reported in 1974 by Farnum, has been utilized as an acceptor unit for organic field-effect transistors (OFETs) and organic photovoltaics (OPVs).^10–15^ Aromatic substituents (such as phenyl or thienyl groups) to the DPP unit are often used to strongly modulate electrical and optical properties. As the DPP unit is strongly electron-withdrawing and forms a planar conjugated backbone, DPP-based polymers have high charge carrier mobilities and small bandgaps.^16–20^ In addition, fluorine atoms have been used in recently developed high-performance D–A polymers.^21^ The strong electronegativity of the fluorine atoms can enhance the oxidative stability of the D–A polymers by lowering the highest occupied molecular orbital (HOMO) energy level. More importantly, fluorine atoms substituted in the conjugated backbone can induce strong interchain interactions through the polar C–F bond dipole and intramolecular interactions with close-lying protons or sulfur atoms in neighboring conjugated units (*i.e.*, through-space H⋯F and S⋯F interactions). These interactions can enhance the orbital overlap between aromatic units, promoting backbone planarity and interchain stacking.^22–24^ We have recently investigated the optical and electrical properties of a DPP-based D–A copolymer (PDPP2DT-F2T2) synthesized using DPP and 3,3′-difluoro-2,2′-bithiophene (F2T2). A comparison with PDPP2DT-T2 synthesized using DPP and 2,2′-bithiophene (T2) units revealed that the F2T2 donor moiety could facilitate a planar polymer backbone conformation and enhance crystallinity and carrier transport.^25^

In this study, we synthesized three D_1_–A–D_2_–A-type DPP-based terpolymers, in which the D_1_ unit was fixed with the F2T2 donor, and D_2_ units were varied using T2, (*E*)-1,2-di(thiophen-2-yl)ethene (TVT), and dithieno[3,2-*b*:2′,3′-*d*]thiophene (DTT). The variation of the second donor moiety enabled the fine-tuning of electronic structures, backbone conformation, and the resulting photovoltaic performance. The structural change caused significant changes in physical properties and photovoltaic performances. In comparison with the PDPP2DT-F2T2 polymer, the PDPPF2T2DPP-T2, PDPPF2T2DPP-TVT, and PDPPF2T2DPP-DTT polymers had high-lying HOMO levels and lower bandgaps. Among PSC devices containing polymer:[6,6]-phenyl-C_71_ butyric acid methyl ester (PC_71_BM) blend films, the performance of the device containing the PDPPF2T2DPP-DTT:PC_71_BM blend film was the best. When diphenyl ether (DPE) was used as a processing additive, photovoltaic performances were further improved. The D_1_–A–D_2_–A-type PDPPF2T2DPP-DTT device exhibited a PCE of 6.35%, which was a great improvement compared with the PCE of the simple D–A-structured PDPP2DT-F2T2:PC_71_BM device. Here, we highlight the advantages of using D_1_–A–D_2_–A polymers (PDPPF2T2DPP-T2, PDPPF2T2DPP-TVT, and PDPPF2T2DPP-DTT) and explain the origin of the difference in photovoltaic property among the PSCs in detail.

## 2. Experimental

### 2.1. Materials

Anhydrous *N*,*N*-dimethylformamide (DMF), anhydrous toluene, anhydrous chloroform, sodium thiosulfate (Na_2_S_2_O_3_), *N*-bromosuccinimide (NBS), and tetrakis(triphenylphosphine)palladium(0) (Pd(PPh_3_)_4_) were purchased from Sigma-Aldrich. Anhydrous acetonitrile was purchased from Alfa Aesar. All the Soxhlet solvents, anhydrous bromine, anhydrous sodium sulfate (Na_2_SO_4_) were purchased from Daejung Chemicals & Metals Co. LTD. (Korea). (3,3′-Difluoro-[2,2′-bithiophene]-5,5′-diyl)bis(trimethylstannane), 2,6-bis(trimethylstannyl)dithieno[3,2-*b*:2′,3′-*d*]thiophene, and (*E*)-1,2-bis(5-(trimethylstannyl)thiophen-2-yl)ethene were purchased from SunaTech Inc. (China). 2,5-Bis(2-decyltetradecyl)-3,6-di(thiophen-2-yl)-2,5-dihydropyrrolo[3,4-*c*]pyrrole-1,4-dione (DPP2DT), 3,6-bis(5-bromothiophen-2-yl)-2,5-bis(2-decyltetradecyl)-2,5-dihydropyrrolo[3,4-*c*]pyrrole-1,4-dione (Br-DPP2DT-Br) and 5,5′-bis(trimethylstannyl)-2,2′-bithiophene were prepared by following the method reported in literature.^26^ Tetrabutylammonium hexafluorophosphate (TBAPF_6_) was purchased for electrochemistry from TCI. CDCl_3_ NMR solvent was purchased from Cambridge Isotope Laboratories. Toluene and DMF, used for polymerization, were separately degassed by freeze–pump–thaw three cycling and added to the reaction mixture. The other solvents were used without further purification.

### 2.2. Synthesis

#### Synthesis of poly(6,6′-(3′′,4′-difluoro-[2,2′:5′,2′′:5′′,2′′′-quaterthiophene]-5,5′′′-diyl)bis(3-(5-bromothiophen-2-yl)-2,5-bis(2-decyltetradecyl)pyrrolo[3,4-*c*]pyrrole-1,4(2*H*,5*H*)-dione)**)**, PDPP2DT-F2T2

Br-DPP2DT-Br (0.2958 g, 0.2614 mmol), (3,3′-difluoro-[2,2′-bithiophene]-5,5′-diyl)bis(trimethylstannane) (0.138 g, 0.2614 mmol), tetrakis(triphenyl-phosphine)palladium(0) (Pd(PPh_3_)_4_) (0.0121 g, 4 mol%) were added to a flame dried one-neck round bottomed flask. Degassed DMF (0.55 mL) and toluene (5.45 mL) were added to the flask and the solution was heated initially to 60 °C with stirring. The reaction temperature was raised to 85 °C gradually at a rate of 1 °C/5 min and after 150 min, gradually at a rate of 1 °C/10 min to 90 °C. After 80 min, the reaction temperature was raised to 100 °C. After 120 min, degassed DMF (0.55 mL) was added to the solution one more time. After 70 min, 2-bromothiophene (0.1 mL) was added to the reaction solution. After stirring for another 1 h, the reaction mixture was cooled to room temperature and transferred to diethylammonium diethyldithiocarbamate (1 M) aqueous solution (50 mL) in 250 mL round bottomed flask using chloroform (20 mL) and stirred at 50 °C for 1 h. The solution was then extracted with chloroform (20 mL × 3), and the chloroform solution was washed with brine and deionized water. The organic solvent was removed by a rotary evaporator to dryness. The crude polymer was redissolved in chloroform (12 mL) and then was precipitated in methanol. The precipitated polymer, collected in a Soxhlet thimble, was purified by Soxhlet extraction using methanol, acetone, hexane and cyclohexane. The cyclohexane fraction was precipitated in methanol, filtered, and dried under vacuum to yield PDPP2DT-F2T2 polymer (0.275 g, 90% yield). Gel-permeation chromatography (GPC) (*o*-dichlorobenzene, 80 °C) *M*_n_ = 57 000 Da, *M*_w_ = 82 000 Da, PDI = 1.44. ^1^H-NMR (300 MHz, CDCl_3_): *δ* = 9.6–8.4 (4H), 5.1–4.8 (4H), 1.55–0.35 (92H).

#### Synthesis of 3-(5-bromothiophen-2-yl)-2,5-bis(2-decyltetradecyl)-6-(thiophen-2-yl)pyrrolo[3,4-*c*]pyrrole-1,4(2*H*,5*H*)-dione, 1

DPP2DT (3.5 g, 3.595 mmol) was dissolved in chloroform (450 mL) and the solution was cooled down to 0 °C in dark condition. NBS (0.7038 g, 3.955 mmol) was added at once and anhydrous DMF (45 mL) was added to the solution and stirred for 30 min. The reaction mixture was warmed up to room temperature and stirred for 40 min. Deionized water (50 mL) was added to the reaction mixture to quench the bromination. Chloroform of the reaction solution was removed by rotary evaporation. The product was then extracted with diethyl ether (30 mL × 5), and the collected organic layer was dried over anhydrous Na_2_SO_4_. The resulting organic layer solvent was evaporated under reduced pressure. The resulting crude product was separated by silica gel column chromatography using dichloromethane and *n*-hexane (1 : 1) mixture as an eluent. The resulting purple solid was recrystallized from dichloromethane/methanol solution to afford pure compound 1 (2.005 g, 53% yield). ^1^H-NMR (300 MHz, CDCl_3_): *δ* = 8.894–8.881 (dd, 1H), 8.623–8.609 (d, 1H), 7.644–7.627 (dd, 1H), 7.282–7.252 (d, 1H), 7.224–7.210 (d, 1H), 4.021–3.995 (d, 2H), 3.946–3.920 (d, 2H), 1.995–1.805 (br, 2H), 1.395–1.112 (br, 80H), 0.898–0.850 (t, 12H). ^13^C-NMR (75 MHz, CDCl_3_): *δ* = 161.67, 161.51, 140.90, 138.96, 135.52, 135.06, 131.36, 131.28, 130.78, 129.78, 128.48, 118.58, 108.17, 107.81, 46.30, 37.79, 37.75, 31.95, 31.19, 30.02, 29.73, 29.70, 29.67, 29.58, 29.39, 26.21, 22.72, 14.15 (see ^1^H- and ^13^C-NMR spectra in Fig. S1 and S2†).

#### Synthesis of 6,6′-(3′′,4′-difluoro-[2,2′:5′,2′′:5′′,2′′′-quaterthiophene]-5,5′′′-diyl)bis(2,5-bis(2-decyltetradecyl)-3-(thiophen-2-yl)pyrrolo[3,4-*c*]pyrrole-1,4(2*H*,5*H*)-dione), 2

Compound 1 (1.3 g, 1.2351 mmol), (3,3′-difluoro-[2,2′-bithiophene]-5,5′-diyl)bis(trimethylstannane) (0.318 g, 0.6025 mmol), tetrakis(triphenyl-phosphine)palladium(0) (Pd(PPh_3_)_4_) (0.0279 g, 0.0241 mmol) were added to a flame dried 250 mL two-neck round bottomed flask. Then, degassed DMF (10 mL) and toluene (50 mL) were added to the flask. The reaction mixture was stirred for 60 min at 110 °C under argon atmosphere. The solvent was removed by rotary evaporator. The reaction mixture was transferred to a 100 mL separatory funnel and diluted with deionized water (50 mL) and extracted with chloroform (30 mL × 3). The collected organic layer was then dried over anhydrous Na_2_SO_4_, and the organic solvent was evaporated under reduced pressure. The crude product was then filtered through a 5 cm silica gel plug using chloroform as an eluent. The collected blue solid was recrystallized from chloroform/isopropyl alcohol solution to afford pure compound 2 (1.22 g, 94.6%). ^1^H-NMR (300 MHz, CDCl_3_): *δ* = 8.915–8.780 (m, 4H), 7.628–7.611 (dd, 2H), 7.335–7.321 (d, 2H), 7.273–7.244 (d, 2H), 7.081 (s, 2H), 4.034–4.015 (d, 8H), 1.930–1.880 (br, 4H), 1.411–1.111 (br, 160H), 0.921–0.788 (m, 24H). ^13^C-NMR (75 MHz, CDCl_3_): *δ* = 161.59, 155.45, 151.94, 140.94, 140.45, 139.14, 136.41, 135.52, 133.30, 130.68, 129.83, 129.23, 128.47, 124.89, 114.32, 113.97, 108.69, 108.09, 46.31, 38.00, 37.78, 31.96, 31.36, 31.22, 30.10, 30.07, 29.72, 29.67, 29.60, 29.40, 26.41, 26.24, 22.72, 14.14. ^19^F-NMR (282 MHz, CDCl_3_): *δ* = −122.05 (see ^1^H-, ^13^C-, and ^19^F-NMR spectra in Fig. S3–S5†).

#### Synthesis of 6,6′-(3′′,4′-difluoro-[2,2′:5′,2′′:5′′,2′′′-quaterthiophene]-5,5′′′-diyl)bis(3-(5-bromothiophen-2-yl)-2,5-bis(2-decyltetradecyl)pyrrolo[3,4-*c*]pyrrole-1,4(2*H*,5*H*)-dione), 3

Compound 2 (1.0 g, 0.4662 mmol) was diluted in anhydrous chloroform (46 mL). Bromine (0.05 mL, 0.979 mmol) was added slowly to the solution. The reaction solution was stirred at room temperature under argon atmosphere. After 1.5 h, saturated Na_2_S_2_O_3_(aq.) solution (20 mL) was added to the reaction solution. After pouring deionized water (50 mL) into the reaction solution, the crude product was extracted with chloroform (4 × 30 mL). The collected organic layer was then dried over anhydrous Na_2_SO_4_. The organic solvent was removed by rotary evaporator and the collected deep blue solid was recrystallized from chloroform/methanol solution to afford pure compound 3 (1.22 g, 99%).^1^H-NMR (300 MHz, CDCl_3_): *δ* = 8.798–8.784 (d, 2H), 8.596–8.582 (d, 2H), 7.261–7.243 (d, 2H), 7.151–7.137 (d, 2H), 7.037 (s, 2H), 3.977–3.924 (dd, 8H), 1.892–1.860 (br, 4H), 1.435–1.110 (br, 160H), 0.901–0.795 (m, 24H). ^13^C-NMR (75 MHz, CDCl_3_): *δ* = 1161.28, 161.13, 155.39, 151.88, 141.24, 139.25, 138.85, 136.32, 135.33, 131.29, 131.23, 129.18, 124.68, 118.98, 114.20, 113.85, 108.37, 108.09, 46.34, 37.88, 37.77, 31.98, 31.41, 31.31, 30.09, 30.07, 29.72, 29.69, 26.63, 26.41, 26.42, 26.27, 22.72, 14.14. ^19^F-NMR (282 MHz, CDCl_3_): *δ* = −122.01 (see ^1^H-, ^13^C-, and ^19^F-NMR spectra in Fig. S6–S8†).

#### Synthesis of poly(3-([2,2′:5′,2′′-terthiophen]-5-yl)-6-(5′′′-(2,5-bis(2-decyltetradecyl)-3,6-dioxo-4-(thiophen-2-yl)-2,3,5,6-tetrahydropyrrolo[3,4-*c*]pyrrol-1-yl)-3′′,4′-difluoro-[2,2′:5′,2′′:5′′,2′′′-quaterthiophen]-5-yl)-2,5-bis(2-decyltetradecyl)pyrrolo[3,4-*c*]pyrrole-1,4(2*H*,5*H*)-dione), PDPPF2T2DPP-T2

Compound 3 (0.300 g, 0.1302 mmol), 5,5′-bis(trimethylstannyl)-2,2′-bithiophene (0.0641 g, 0.1302 mmol), tetrakis(triphenyl-phosphine)palladium(0) (Pd(PPh_3_)_4_) (0.006 g, 4 mol%) were added to a flame dried one-neck round bottomed flask. Degassed DMF (0.9 mL) and toluene (4.4 mL) were added to the flask and the solution was heated initially at 60 °C with stirring. The reaction temperature was then raised to 90 °C gradually at a rate of 1 °C/150 s. After 18 min, the reaction solution became a gel state. The reaction temperature was quickly cooled down to 60 °C and chloroform (5 mL) was added to the reaction mixture. After vigorously stirring for 1 h, the reaction mixture was transferred to diethylammonium diethyldithiocarbamate (1 M) aqueous solution (50 mL) in 250 mL round bottomed flask using hot chloroform (20 mL) and stirred at 50 °C for 1 h. The polymer was extracted with chloroform (30 mL × 3). The collected organic layer was then washed brine and deionized water. After removing organic solvent under reduced pressure, the crude polymer was dissolved in chloroform (15 mL) and then was precipitated in methanol (300 mL). The collected polymer was further purified by Soxhlet extraction using methanol, acetone, hexane, cyclohexane, dichloromethane, and chloroform. The chloroform fraction was precipitated in methanol, filtered, and dried under vacuum to yield PDPPF2T2DPP-T2 polymer (0.284 g, 94.6% yield). Gel-permeation chromatography (GPC) (*o*-dichlorobenzene, 80 °C) *M*_n_ = 129 000 Da, *M*_w_ = 276 000 Da, PDI = 2.14. ^1^H-NMR (300MHz, CDCl_3_): *δ* = 9.8–8.5 (8H), 7.25–6.1 (6H), 5.2–4.7(8H), 1.55–0.35 (184H) (see ^1^H-NMR spectra in Fig. S9†).

#### Synthesis of poly((*E*)-3-(5′′′-(2,5-bis(2-decyltetradecyl)-3,6-dioxo-4-(5′-(2-(thiophen-2-yl)vinyl)-[2,2′-bithiophen]-5-yl)-2,3,5,6-tetrahydropyrrolo[3,4-*c*]pyrrol-1-yl)-3′′,4′-difluoro-[2,2′:5′,2′′:5′′,2′′′-quaterthiophen]-5-yl)-2,5-bis(2-decyltetradecyl)-6-(thiophen-2-yl)pyrrolo[3,4-*c*]pyrrole-1,4(2*H*,5*H*)-dione), PDPPF2T2DPP-TVT

Compound 3 (0.300 g, 0.1302 mmol), (*E*)-1,2-bis(5-(trimethylstannyl)thiophen-2-yl)ethene (0.0675 g, 0.1302 mmol), tetrakis(triphenyl-phosphine)palladium(0) (Pd(PPh_3_)_4_) (0.006 g, 4 mol%) were added to a flame dried one-neck round bottomed flask. Degassed DMF (0.9 mL) and toluene (8.8 mL) were added to the flask. The reaction solution was heated initially to 60 °C with stirring. The reaction temperature was raised to 90 °C gradually at a rate of 1 °C/150 s. After 2 h, 2-bromothiophene (0.1 mL) was added to the solution. The reaction solution was then stirred for another 1 h and cooled down to room temperature. After diluting the reaction solution with chloroform (30 mL), the reaction mixture was transferred to diethylammonium diethyldithiocarbamate (1 M) aqueous solution (50 mL) 250 mL round bottomed flask and stirred at 50 °C for 1 h. The solution was extracted with chloroform (30 mL × 3). The collected organic layer was then washed brine and deionized water. After removing organic solvent under reduced pressure, the crude polymer was dissolved in chloroform (16 mL) and then was precipitated in methanol (300 mL). The collected polymer was further purified by Soxhlet extraction using methanol, acetone, hexane, and cyclohexane. The cyclohexane fraction was precipitated in methanol, filtered, and dried under vacuum to yield PDPPF2T2DPP-TVT polymer (0.2519 g, 82.9% yield). Gel-permeation chromatography (GPC) (*o*-dichlorobenzene, 80 °C) *M*_n_ = 22 000 Da, *M*_w_ = 119 000 Da, PDI = 5.31. ^1^H-NMR (300MHz, CDCl_3_): *δ* = 9.3–8.4 (8H), 7.25–5.9 (6H), 5.1–4.6(8H), 1.58–0.36 (184H) (see ^1^H-NMR spectra in Fig. S10†).

#### Synthesis of poly(3-(5′′′-(2,5-bis(2-decyltetradecyl)-3,6-dioxo-4-(thiophen-2-yl)-2,3,5,6-tetrahydropyrrolo[3,4-*c*]pyrrol-1-yl)-3′′,4′-difluoro-[2,2′:5′,2′′:5′′,2′′′-quaterthiophen]-5-yl)-2,5-bis(2-decyltetradecyl)-6-(5-(dithieno[3,2-*b*:2′,3′-*d*]thiophen-2-yl)thiophen-2-yl)pyrrolo[3,4-*c*]pyrrole-1,4(2*H*,5*H*)-dione), PDPPF2T2DPP-DTT

Compound 3 (0.300 g, 0.1302 mmol), 2,6-bis(trimethylstannyl)dithieno[3,2-*b*:2′,3′-*d*]thiophene (0.068 g, 0.1302 mmol), tetrakis(triphenyl-phosphine)palladium(0) (Pd(PPh_3_)_4_) (0.006 g, 4 mol%) were added to a flame dried one-neck round bottomed flask. Degassed DMF (1.1 mL) and toluene (5.5 mL) were added to the flask and the solution was heated initially to 60 °C with stirring. The reaction temperature was raised to 90 °C gradually at a rate of 1°C/5 min. After 6 min, the reaction solution became a gel state. The reaction temperature was cooled down to 60 °C and chloroform (5 mL) was added was added to the reaction mixture. After vigorous stirring for 1 h, the reaction mixture was transferred to diethylammonium diethyldithiocarbamate (1 M) aqueous solution (50 mL) in 250 mL round bottomed flask using hot chloroform (30 mL) and stirred at 50 °C for another 1 h. The polymer solution was extracted with chloroform (30 mL × 3). The collected organic layer was then washed brine and deionized water. After removing organic solvent under reduced pressure, the crude polymer was dissolved in chloroform (21 mL) and then was precipitated in methanol (300 mL). The collected polymer was further purified by Soxhlet extraction using methanol, acetone, hexane, cyclohexane, dichloromethane, and chloroform. The chloroform fraction was precipitated in methanol, filtered, and dried under vacuum to yield PDPPF2T2DPP-DTT polymer (0.2819 g, 92.6% yield). Gel-permeation chromatography (GPC) (*o*-dichlorobenzene, 80 °C) *M*_n_ = 186 000 Da, *M*_w_ = 627 000 Da, PDI = 3.37. ^1^H-NMR (300MHz, CDCl_3_): *δ* = 9.5–8.2 (8H), 7.24–5.8 (4H), 5.2–4.7(8H), 1.53–0.36 (184H) (see ^1^H-NMR spectra in Fig. S11†).

### 2.3. Characterizations and measurements

To identify the molecular structures of all the synthesized products, ^1^H-NMR, ^13^C-NMR and ^19^F-NMR spectra were taken on Bruker Avance III 300MHz. Average molecular weights and polydispersity index (PDI) of synthesized polymers were determined by an Agilent GPC system (GPC 1200 system) at 80 °C. *o*-Dichlorobenzene was an eluent, and polystyrene standards were used for molecular weight calibration. UV-visible absorption spectra were taken on Agilent 8453 UV-Vis spectrophotometer. Differential scanning calorimetry (DSC) data were obtained on DSC Q2000 differential scanning calorimeter from TA instruments. Heating and cooling temperature was scanned at a rate of 10 °C min^−1^. Thermogravimetric analysis (TGA) data were obtained on TGA Q50 under N_2_ atmosphere at a temperature scan rate of 25 °C min^−1^. Cyclic voltammetry (CV) was performed using a CorrTest instruments and 0.1 M tetrabutylammonium hexafluorophosphate (TBAPF_6_) solution in anhydrous acetonitrile was prepared as an electrolyte. Pt wires were used as the counter electrode and working electrode. Synthesized polymers were coated on the Pt wire working electrode; Ag wire was used as a reference electrode. Ferrocene (Fc) was used as the internal standard; ferrocene oxidation (Fc/Fc^+^) potential was assumed to be −4.8 eV. The voltage sweep rate was 50 mV s^−1^. HOMO levels were determined from the onsets of the anodic curves. Density functional theory (DFT) calculations was conducted as follows: HOMO and LUMO energies were calculated by quantum calculation as implemented in Gaussian 09 package. Geometry optimization, single point calculation and frequency analysis were performed using density functional theory (B3LYP method) with 6-311G(d) basis set. In order to obtain the representative structure of the target molecules, the global minimum structures were searched in two steps. Firstly, the optimized structures of fragment molecules consisting of two different residues were determined by the calculated potential energy surface as a function of dihedral angle between residues. The model molecules for the investigated polymers was built based on the optimized structure of the fragment molecule. Tetramer, dimer, dimer and dimer for PDPP2DT-F2T2, PDPPF2T2DPP-T2, PDPPF2T2DPP-TVT and PDPPF2T2DPP-DTT, respectively were chosen and the bulky decyltetradecyl groups were replaced with methyl groups to reduce computational cost. The model molecules were freely optimized and confirmed by frequency analysis. The global minimum structures were used to determine the HOMO and LUMO energies. GIWAXS measurements were accomplished at PLS-II 9A U-SAXS beamline of the Pohang Accelerator Laboratory in Republic of Korea. X-rays coming from the in-vacuum undulator (IVU) were monochromated (*E*_k_ = 11.065 keV, wavelength *λ* = 1.10994 Å) using a Si(111) double crystal monochromator and focused both horizontally and vertically at the sample position (450 (H) × 60 (V) μm^2^ in FWHM@sample position) using K–B type mirrors system. GIWAXS sample stage was equipped with a 7-axis motorized stage for the fine alignment of sample, and the incidence angle of X-ray beam was set to be 0.12°–0.14° for polymer films and polymer:PC_71_BM blend films. GIWAXS patterns were recorded with a 2D CCD detector (Rayonix SX165) and X-ray irradiation time was 6–9 s, dependent on the saturation level of the detector. Diffraction angles were calibrated using a sucrose standard (Monoclinic, *P*2_1_, *a* = 10.8631 Å, *b* = 8.7044 Å, *c* = 7.7624 Å, *β* = 102.938°) and the sample-to-detector distance was ∼231 mm. Samples for GIWAXS measurements were prepared by spin-coating polymers and polymer:PC_71_BM blend solutions with various ratios on top of the PEDOT:PSS coated Si wafer substrates. AFM characterization: an Agilent 5500 scanning probe microscope (SPM) running with a Nanoscope V controller was used to obtain AFM images of polymer:PC_71_BM blend thin films. AFM images were recorded in high-resolution tapping mode under ambient conditions. Premium silicon cantilevers (TESP-V2) were used with a rotated tip to provide more symmetric representation of features over 200 nm.

### 2.4. Device fabrication

Glass/patterned indium tin oxide (ITO) substrates were cleaned with detergent and ultra-sonicated in deionized water, acetone, and isopropyl alcohol sequentially and dried in an oven for 12 h. The substrates were subjected to UV/ozone treatment for 15 min and then poly(3,4-ethylenedioxythiophene):polystyrene sulfonic acid (PEDOT:PSS, CLEVIOS P VP Al4083) was spin-coated on top of the cleaned ITO substrates through 0.45 μm cellulose acetate syringe filter and were dried at 140 °C to remove moisture. The substrates were transferred to globe box filled in N_2_ and photoactive solution was spin-cast. These polymers (12 mg mL^−1^) were blended with PC_71_BM with various ratio in chlorobenzene. 100 nm thick Al electrode was evaporated on top of the photoactive layer under high vacuum (<10^−6^ torr) through a mask. The active area of the device is 3.67 mm^2^. The photovoltaic characteristics were measured in glove box by using a high quality optical fiber to guide light from the solar simulator. The current density–voltage (*J*–*V*) characteristics of the PSC were measured using a Keithley 2635A source measurement unit under AM 1.5G illumination at 100 mW cm^−2^. The incident photon-to-current efficiency (IPCE) was measured by using a QEX7 from PV measurement Inc. For light intensity dependence measurement, the devices were placed under a solar simulator by using a set of neutral density filters. Neutral density filters have the ability to block a certain amount of the light, which reduces the intensity.

## 3. Results and discussion

### 3.1. Polymer synthesis and thermal properties

**Scheme 1:**
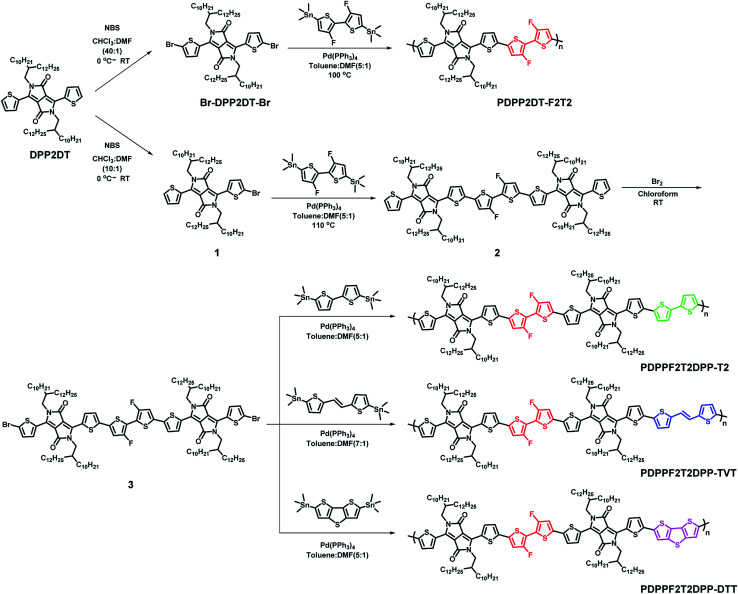
Synthetic routes for the DPP-based regio-regular terpolymers.

The synthetic routes for all of the DPP-based terpolymers as well as the PDPP2DT-F2T2 polymer are shown in Scheme 1. To synthesize the terpolymers, compound 3, a key monomer, was prepared *via* three steps: monobromination of DPP2DT, Stille coupling with 2,6-bis(trimethylstannyl)dithieno[3,2-*b*:2′,3′-*d*]thiophene compound, and dibromination. Polymers were prepared *via* the Stille polymerization of an acceptor monomer (Br-DPP2DT-Br or Br-DPPF2T2DPP-Br) and a bis(trimethylstannyl) donor monomer (F2T2, T2, TVT, or DTT) using Pd(PPh_3_)_4_ catalyst in a toluene:DMF cosolvent system. The crude polymer solutions were treated with 1 M diethylammonium diethyldithiocarbamate solution to remove the Pd-catalyst, precipitated in methanol, and further purified by the Soxhlet extraction method. The final fraction was precipitated and dried. The resulting polymers were obtained with a high yield (up to 83%) and were highly soluble in organic solvents like chloroform, toluene, and chlorobenzene (CB). Thermal stability was determined by thermogravimetric analysis (TGA) and differential scanning calorimetry (DSC). TGA results revealed that all terpolymers had a decomposition temperature (*T*_d_) of > 400 °C, while DSC data showed no noticeable transitions (see Fig. S12†).

### 3.2. Optical and electrochemical properties

**Fig. 1:**
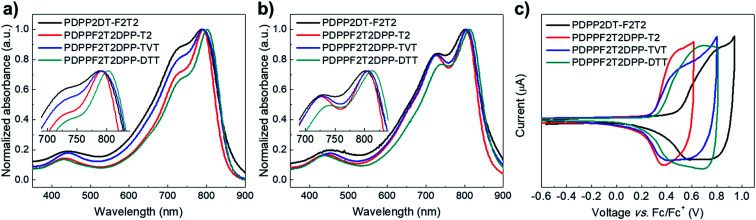
(a) UV-visible absorption spectra of the polymer solutions of chlorobenzene. (b) UV-visible absorption spectra of the polymer films. (c) Cyclic voltammograms of the polymer films.

Optical and electrochemical properties of the synthesized polymers were summarized in the Fig. 1 and Table 1. Fig. 1a and b shows that all of the synthesized polymers had dual absorption regions at 400–500 nm and 700–800 nm, which are attributed to two different π–π transitions. The vibronic peaks in the 700–800 nm region were more pronounced in the film state (Fig. 1b) than in solution (Fig. 1c). The maximum absorption peaks in the solution were slightly more red-shifted in the film state (PDPP2DT-F2T2: 791 nm → 799 nm, PDPPF2T2DPP-T2: 791 nm → 801 nm, PDPPF2T2DPP-TVT: 791 nm → 803 nm, PDPPF2T2DPP-DTT: 803 nm → 810 nm). This finding suggests that polymer aggregation was increased in the film state. In particular, the PDPPF2T2DPP-DTT polymer had not only a red-shifted absorption peak but also a pronounced vibronic peak at 810 nm, indicating that the PDPPF2T2DPP-DTT polymer significantly improved interchain interactions in the film state. Optical bandgaps (*E*^opt^_g_s) were estimated from the absorption onsets of the polymer films, which were 1.40, 1.43, 1.42, and 1.41 eV for PDPP2DT-F2T2, PDPPF2T2DPP-T2, PDPPF2T2DPP-TVT, and PDPPF2T2DPP-DTT, respectively.

**Table 1:** Molecular weight and optical and electrochemical properties of the polymers

Polymer	*M*_n_ (kDa)	PDI	*λ*_peak_ (nm)		*λ*_onset_ a (nm)	HOMO^b^ (eV)	LUMO^c^ (eV)	*E* ^opt^_g_ d (eV)
			Solution	Film				
PDPP2DT-F2T2	57 000	1.4	790	799	884	−5.38	−3.90	1.48
PDPPF2T2DPP-T2	129 000	2.1	791	801	865	−5.10	−3.67	1.43
PDPPF2T2DPP-TVT	22 000	5.3	791	803	876	−5.08	−3.66	1.42
PDPPF2T2DPP-DTT	186 000	3.4	803	810	881	−5.13	−3.72	1.41

a Determined from polymer films.

b Determined using an ionization potential of −4.8 eV for Fc/Fc^+^.

c Determined as *E*_HOMO_ + *E*^opt^_g_.

d Determined from the absorption onsets (*λ*_onset_).

Cyclic voltammetry (CV) measurements were conducted to investigate the electrochemical properties of the synthesized polymers. Fig. 1c shows the cyclic voltammograms of the polymer films, which were measured in a 0.1 M tetrabutylammonium hexafluorophosphate (TBAPF_6_) solution of anhydrous acetonitrile. The onsets of oxidation potential for PDPP2DT-F2T2, PDPPF2T2DPP-T2, PDPPF2T2DPP-TVT, and PDPPF2T2DPP-DTT were 0.49, 0.30, 0.28, and 0.33 V, corresponding to −5.38, −5.10, −5.08, and −5.13 eV of HOMO energy levels, respectively. Fluorine atoms in the PDPP2DT-F2T2 effectively lowered the HOMO level of the PDPP2DT-T2 polymer that has the same chemical structure except fluorine atoms,^25^ whereas electron-donating units (T2, TVT, and DTT moieties) slightly increased the HOMO levels compare to that of the PDPP2DT-F2T2 polymer. LUMO levels were −3.90, −3.67, 3.66, and −3.72 eV (estimated by the equation: *E*_HOMO_ + *E*^opt^_g_) for PDPP2DT-F2T2, PDPPF2T2DPP-T2, PDPPF2T2DPP-TVT, and PDPPF2T2DPP-DTT, respectively.

### 3.3. Density functional theory (DFT) calculations

**Fig. 2:**
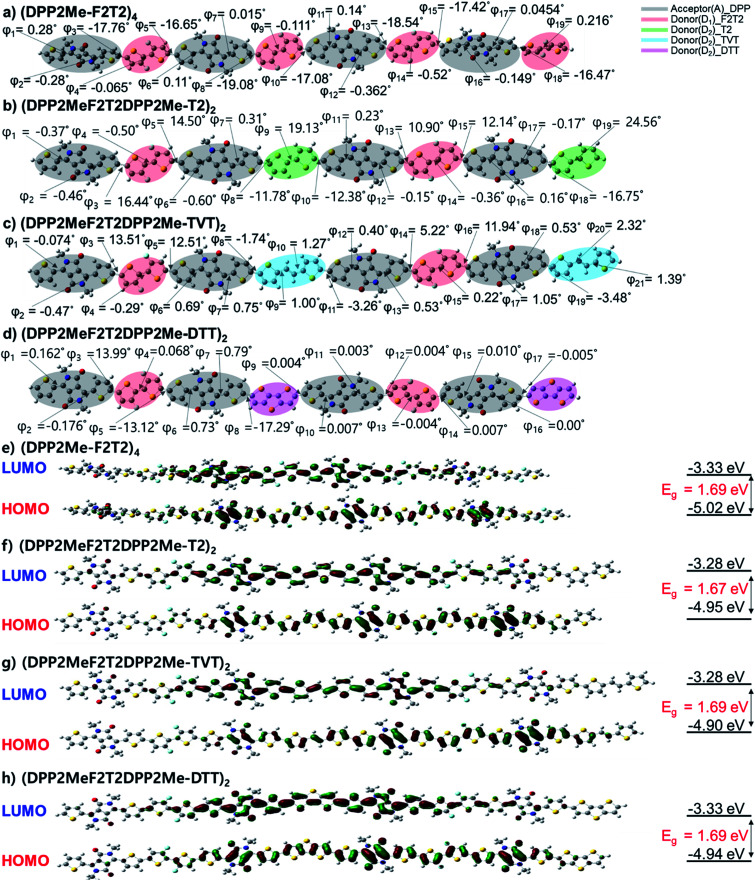
(a–d) Optimized geometry of (DPP2Me-F2T2)_4_, (DPP2MeF2T2DPP2Me-T2)_4_, (DPP2MeF2T2DPP2Me-TVT)_4_ and (DPP2MeF2T2DPP2Me-DTT)_4_. (e–h) Surface plots and energy levels of the frontier orbitals of (DPP2Me-F2T2)_4_, (DPP2MeF2T2DPP2Me-T2)_4_, (DPP2MeF2T2DPP2Me-TVT)_4_ and (DPP2MeF2T2DPP2Me-DTT)_4_.

Molecular backbone geometries and electronic properties were evaluated using Gaussian 09 program package at the DFT level (B3LYP, 6-311G(d)). The model molecules of the polymers were (DPP2Me-F2T2)_4_, (DPP2MeF2T2DPP2Me-T2)_2_, (DPP2MeF2T2DPP2Me-TVT)_2_, and (DPP2MeF2T2DPP2Me-DTT)_2_, where the long 2-decyltetradecyl groups were superseded by methyl groups. The energy-minimized molecular geometries of the model molecules are shown in Fig. 2a–d. The dihedral angles (DAs) were examined. The DA between two fluorothiophene rings in the F2T2 unit was almost 0° through S⋯F interaction; however, there was some degree of distortion (17–19°) between the F2T2 units and neighboring thiophenes due to steric hindrance in the (DPP2Me-F2T2)_4_ structure. When the F2T2 units were replaced with the T2, TVT, and DTT units, the DA values between the F2T2 unit and the neighboring thiophene were decreased to 13–16° for the left half structure or almost 0° for the right half structure, and the planarity was improved in the order of T2, TVT, and DTT. In addition, the replacement of the F2T2 units with T2 units increased the DAs in the bithiophene regions corresponding to the F2T2 units, whereas the DAs between thiophene rings connected to the DPP moiety and T2 units were reduced compared with those of the F2T2-based structure, *i.e.*, (DPP2Me-F2T2)_4_. Accordingly, the overall twists of (DPP2MeF2T2DPP2Me-T2)_2_ appeared to be similar to those of (DPP2Me-F2T2)_4_. On the other hand, the additional planar moieties of the TVT and DTT units resulted in more planar structures. In comparison with the UV-visible absorption spectra of the polymers in the solution state (Fig. 1a), the contribution of DA differences was minor because the *λ*_peak_ values were nearly the same. This result is consistent with previous studies reporting that DA values under 30° do not significantly alter electronic properties.^27^ However, the high coplanarity greatly affected interchain interactions in the film state (Fig. 1b), especially for the (DPP2MeF2T2DPP2Me-DTT)_2_ structure.

Next, HOMO and LUMO surface plots and energy levels of the model molecules were examined. In both the HOMO and LUMO orbitals, electronic densities were well distributed over the backbone for all of the model molecules. HOMO levels were −5.02, −4.95, −4.90, and −4.94 eV and LUMO levels were −3.33, −3.28, −3.28 and −3.33 eV for (DPP2Me-F2T2)_4_, (DPP2MeF2T2DPP2Me-T2)_2_, (DPP2MeF2T2DPP2Me-TVT)_2_, and (DPP2MeF2T2DPP2Me-DTT)_2_, respectively. The bandgaps (*E*_g_s) were 1.69, 1.67, 1.61, and 1.62 eV for (DPP2Me-F2T2)_4_, (DPP2MeF2T2DPP2Me-T2)_2_, (DPP2MeF2T2DPP2Me-TVT)_2_, and (DPP2MeF2T2DPP2Me-DTT)_2_, respectively. The HOMO level of the most F-rich molecule, (DPP2Me-F2T2)_4_, was the lowest among the terpolymer model molecules, consistent with CV data and our expectation. This result indicated the significance of fluorine atoms in changing molecular electronic levels.

### 3.4. Photovoltaic characteristics

**Fig. 3:**
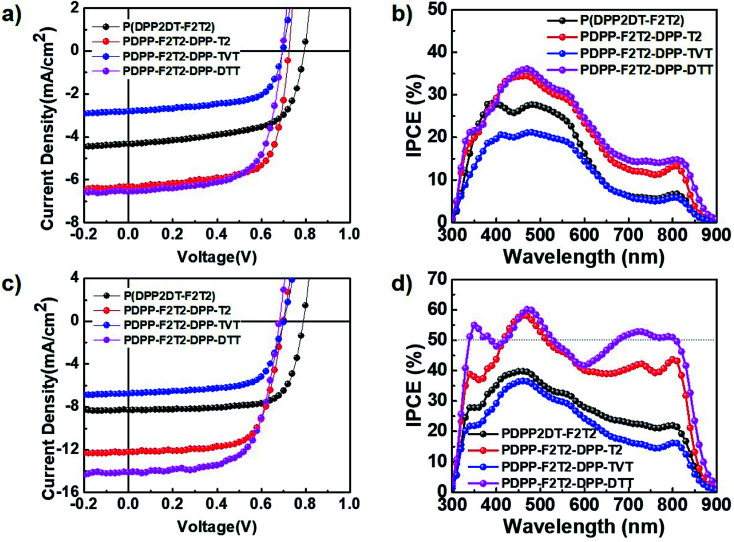
(a) *J*–*V* characteristics and (b) IPCE spectra of polymer:PC_71_BM PSCs without DPE use. (c) *J*–*V* characteristics and (d) IPCE spectra of polymer:PC_71_BM PSCs with DPE use.

We investigated the photovoltaic characteristics of PSC devices containing the DPP-based polymers under simulated AM 1.5G sunlight (100 mW cm^−2^) with a conventional structure (ITO/PEDOT:PSS/polymer:PC_71_BM/Al). To optimize the polymer:PC_71_BM blend ratio, blend films were processed using pure CB or CB containing 3 vol% DPE as the solvent. The optimal polymer:PC_71_BM ratios were 1 : 2, 1 : 3, 1 : 3, and 1 : 2 (w/w) for the PDPP2DT-F2T2, PDPPF2T2DPP-T2, PDPPF2T2DPP-TVT, and PDPPF2T2DPP-DTT polymers, respectively. The device current–voltage (*J*–*V*) characteristics and IPCE spectra of each optimized device are shown in Fig. 3, and the device photovoltaic parameters are summarized in Table 2. The PDPP2DT-F2T2 device had the highest *V*_oc_ (0.79 V). This high *V*_oc_, one of the highest *V*_oc_ values among the DPP polymer-based PSCs, may be correlated with the lower-lying HOMO level of the PDPP2DT-F2T2 film due to the high number of F atom substituents in the conjugated backbone. This can be confirmed by comparison with PSCs containing PDPPF2T2DPP-T2 (F atom substitution in the alternate quarter thiophene bridges) and DT-PDPP4T^14^ (no F atom substitution in the quarter thiophene bridges), which had *V*_oc_ values of 0.70 and 0.64 V, respectively. In comparison with devices containing PDPP2DT-F2T2 and PDPPF2T2DPP-TVT, devices containing PDPPF2T2DPP-T2 and PDPPF2T2DPP-DTT demonstrated a higher performance with a maximum PCE of 3.20 and 3.01%, *J*_sc_ of 6.32 and 6.59 mA cm^−2^, *V*_oc_ of 0.70 and 0.69 V, and FF of 0.70 and 0.66, respectively.

**Table 2:** Summary of photovoltaic characteristics for polymer:PC_71_BM blends

Polymer	Ratio	Additive	*J*_sc_ [mA cm^−2^]	Cal. *J*_sc_ [mA cm^−2^]	*V*_oc_ [V]	FF	PCE [%]
PDPP2DT-F2T2	1 : 2	0 vol% DPE	4.33	4.55	0.79	0.63	2.16 (1.91 ± 0.24)
PDPPF2T2DPP-T2	1 : 3		6.32	6.31	0.70	0.70	3.20 (2.89 ± 0.31)
PDPPF2T2DPP-TVT	1 : 3		3.75	3.72	0.70	0.67	1.77 (1.49 ± 0.27)
PDPPF2T2DPP-DTT	1 : 2		6.59	6.91	0.69	0.66	3.01 (2.78 ± 0.22)
PDPP2DT-F2T2	1 : 2	3 vol% DPE	8.29	8.43	0.79	0.73	4.78 (4.53 ± 0.24)
PDPPF2T2DPP-T2	1 : 3		12.20	12.14	0.70	0.69	5.89 (5.70 ± 0.19)
PDPPF2T2DPP-TVT	1 : 3		6.76	7.01	0.70	0.68	3.20 (3.04 ± 0.15)
PDPPF2T2DPP-DTT	1 : 2		14.14	15.15	0.68	0.66	6.35 (6.14 ± 0.20)

Processing additives can be used to modify the morphology of the photoactive layer, greatly improving device performance.^28–30^ Following optimization with various processing additives, DPE was found to be suitable for our DPP polymer-based PSCs. As shown in Fig. 3 and Table 2, the addition of 3 vol% DPE to CB led to a marked enhancement in all device parameters. In particular, the PCEs were significantly increased from 2.18, 3.20, 1.77, and 3.01% to 4.78, 5.89, 3.20, and 6.35% for devices containing PDPP2DT-F2T2, PDPPF2T2DPP-T2, PDPPF2T2DPP-TVT, and PDPPF2T2DPP-DTT, respectively. High FF values (0.66–0.73) were obtained for all devices, and *J*_sc_ values were a main factor for determining differences in PCE values. The optimized thicknesses of photoactive layers processed with the DPE additive were 100–170 nm. Among four devices containing DPP polymer-based PSCs processed with the CB:DPE solvent, the PDPPF2T2DPP-DTT device had the best PCE (6.35%) with a *J*_sc_ of 14.14 mA cm^−2^, *V*_oc_ of 0.68 V, and FF of 0.66. The PDPPF2T2DPP-DTT device had the highest IPCE values in the whole wavelength region (300–900 nm) as well. In particular, the PDPPF2T2DPP-DTT device had IPCE values of over 50% in the long wavelength range (675–810 nm), indicating that PDPPF2T2DPP-DTT has great potential for use in high-performance tandem PSCs as a component such as a front or back cell.

### 3.5. Charge transport characteristics

**Fig. 4:**
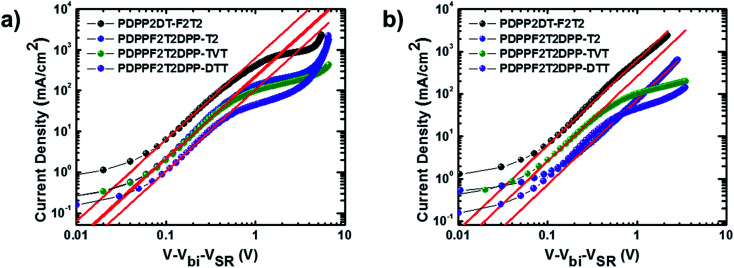
Measured *J*–*V* characteristics with red lines fitted by SCLC model for (a) hole-only devices and (b) electron-only devices containing the polymer:PC_71_BM blend films.

Vertical charge transport characteristics in the photoactive films was evaluated using a space charge limited current (SCLC) model. Hole-only (ITO/PEDOT:PSS/polymer:PC_71_BM/Au) and electron-only (fluorine-doped tin oxide (FTO)/polymer:PC_71_BM/Al) devices were fabricated under optimized device fabrication conditions. To ensure accuracy in carrier mobility estimation, the potential loss due to the series resistance of the ITO and the built-in potential were taken into consideration. *J*–*V* characteristics (Fig. 4) showed a quadratic dependence on voltage over a range of several volts, which was consistent with the Mott–Gurney equation:^31,32^*J*_SCL_ = 9*ε*_0_*ε*_r_*μV*^2^/8*L*^3^where *ε*_0_ is the free-space permittivity, *ε*_r_ is the dielectric constant of the polymer:PC_71_BM blend films, *μ* is the mobility, *V* is the applied voltage and *L* is the thickness of the photoactive films. As shown in Table 3, the hole (*μ*_h_) mobilities were 1.08 × 10^−3^, 3.19 × 10^−3^, 7.03 × 10^−4^, and 1.74 × 10^−3^ cm^2^ V^−1^ s^−1^, and the electron (*μ*_e_) mobilities were 2.75 × 10^−3^, 4.21 × 10^−3^, 2.19 × 10^−3^, and 2.17 × 10^−3^ cm^2^ V^−1^ s^−1^ for the polymer:PC_71_BM devices containing PDPP2DT-F2T2, PDPPF2T2DPP-T2, PDPPF2T2DPP-TVT, and PDPPF2T2DPP-DTT, respectively. All of the devices were efficient in the vertical transport of charge carriers with a mobility of 10^−3^ cm^2^ V^−1^ s^−1^. The *μ*_h_/*μ*_e_ ratios were 0.393, 0.758, 0.321, and 0.803 for polymer:PC_71_BM devices containing PDPP2DT-F2T2, PDPPF2T2DPP-T2, PDPPF2T2DPP-TVT, and PDPPF2T2DPP-DTT, respectively. The photoactive blends with PDPPF2T2DPP-T2 and PDPPF2T2DPP-DTT had the most balanced *μ*_h_/*μ*_e_ ratio (0.758–0.803), suggesting efficient charge transport and extraction with minimal electron–hole recombination in the PSC device. On the contrary, the poor photovoltaic properties of the devices containing the PDPP2DT-F2T2:PC_71_BM and PDPPF2T2DPP-TVT:PC_71_BM blend films may be mainly attributed to the less balanced charge carrier transport, the build-up of space charges, and the resulting electron–hole recombination. Moreover, when the carrier mobilities of pristine polymer films (Fig. S13† and Table S1†) were compared with those of the polymer:PC_71_BM blend films, the hole mobilities in the blend films were decreased slightly whereas the electron mobilities were improved significantly because of using PC_71_BM.

**Table 3:** Hole and electron mobilities of polymer:PC_71_BM blend films, estimated using the SCLC method

Polymer	*μ*_h_ [cm^2^ V^−1^ s^−1^]	Thickness (hole)	*μ*_e_ [cm^2^ V^−1^ s^−1^]	Thickness (electron)	*μ*_h_/*μ*_e_
PDPP2DT-F2T2	1.08 × 10^−3^	80	2.75 × 10^−3^	110	0.393
PDPPF2T2DPP-T2	3.19 × 10^−3^	160	4.21 × 10^−3^	120	0.758
PDPPF2T2DPP-TVT	7.03 × 10^−4^	100	2.19 × 10^−3^	120	0.321
PDPPF2T2DPP-DTT	1.74 × 10^−3^	170	2.17 × 10^−3^	180	0.802

**Fig. 5:**
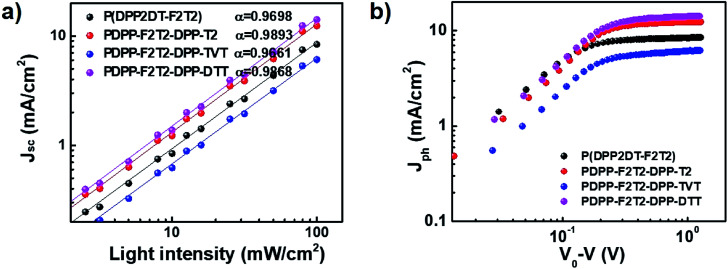
Plots of measured (a) *J*_sc_*versus* light intensity and (b) photocurrent density (*J*_ph_) *versus* effective voltage.

To gain insights on the charge recombination and charge extraction of PSCs containing the terpolymers, we examined the light intensity dependence of *J*–*V* characteristics under short-circuit conditions. Fig. 5a shows a log–log plot of *J*_sc_ as a function of the light intensity. The *J*–*V* curves were fitted according to the power-law dependence of *J*_sc_ on the light intensity:*J*_sc_ ∝ *I*^*α*^where *I* is the light intensity, and *α* is the exponent constant for polymer:PC_71_BM-based PSCs. The *α* values were 0.9698, 0.9893, 0.9661, and 0.9868 for the devices containing the PDPP2DT-F2T2, PDPPF2T2DPP-T2, PDPPF2T2DPP-TVT, and PDPPF2T2DPP-DTT polymers, respectively. The devices containing PDPPF2T2DPP-T2 and PDPPF2T2DPP-DTT had *α* values closer to 1, indicating little bimolecular recombination under short circuit conditions. The device containing PDPPF2T2DPP-TVT had a relatively lower *α* value, indicating a relatively larger degree of bimolecular recombination due to a poorer morphology (see below) and lower charge transport. In addition, we examined the dependence of the net photocurrent (*J*_ph_) on the effective voltage (*V*_eff_) (Fig. 5b). *J*_ph_ is the difference between the current density under illumination and dark conditions of the PSCs (*J*_ph_ = *J*_L_ − *J*_D_). *V*_eff_ is the difference between the compensation voltage (*V*_0_) at *J*_ph_ = 0 and applied bias voltage (*V*), *i.e.*, *V*_eff_ = *V*_0_ − *V*. At a high *V*_eff_, the photocurrent was saturated without recombination, demonstrating that all photo-generated charges were collected at the electrodes. The devices containing PDPPF2T2DPP-T2 and PDPPF2T2DPP-DTT had the highest *J*_ph_s, whereas those containing PDPP2DT-F2T2 and PDPPF2T2DPP-TVT had a substantially lower *J*_ph_s. With increasing *V*_eff_, the photocurrent gradually became saturated for the blend films with PDPP2DT-F2T2, PDPPF2T2DPP-T2, and PDPPF2T2DPP-DTT at *V*_eff_ = ∼0.80 V; however, the blend film with PDPPF2T2DPP-TVT did not exhibit a saturation region over 1 V. The devices containing PDPP2DT-F2T2, PDPPF2T2DPP-T2, and PDPPF2T2DPP-DTT had high *J*_ph_/*J*_sat_ ratios (96%, 99%, and 99%, respectively), where the ratio of *J*_ph_ to *J*_sat_ is the product of charge dissociation and collection probabilities. However, the device containing PDPPF2T2DPP-TVT had a slightly lower *J*_ph_/*J*_sat_ ratio (89%) due to the high bimolecular recombination. These results are in good agreement with the mobility and light intensity-dependent *J*_sc_ data.

### 3.6. Morphological characteristics

**Fig. 6:**
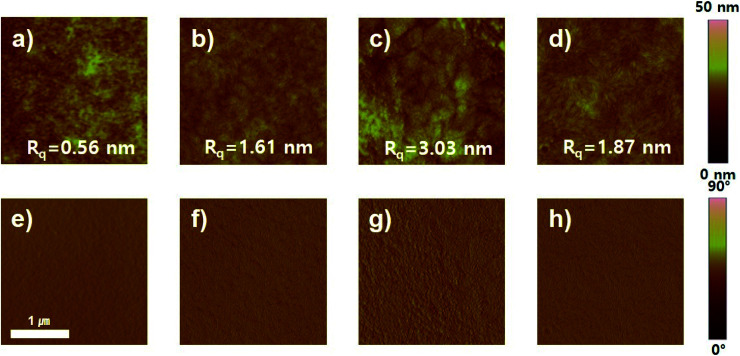
Surface morphology of polymer:PC_71_BM blend films with DPE use. AFM (a–d) topographic and (e–h) phase images: (a and e) PDPP2DT-F2T2, (b and f) PDPPF2T2DPP-T2, (c and g) PDPPF2T2DPP-TVT, and (d and h) PDPPF2T2DPP-DTT.

To analyze the improvement in device performance caused by the use of the DPE additive, we examined the polymer:PC_71_BM blend films by atomic force microscopy (AFM), field-emission transmission electron microscopy (FE-TEM), and two-dimensional grazing incidence wide-angle X-ray scattering (GIWAXS). According to AFM results, the topography and phase images of photoactive films with and without DPE addition showed a clearly different morphology (Fig. 6 and S14†). The AFM topographic images of the polymer:PC_71_BM blend films prepared with the CB and CB:DPE solvents were examined. All photoactive blend films without DPE addition had high root-mean-square (rms) roughness values of 3.16–12.17 nm. In contrast, the blend films with DPE addition had a significantly different morphology. Among the photoactive films, the PDPPF2T2DPP-T2:PC_71_BM and PDPPF2T2DPP-DTT:PC_71_BM blend films prepared with the CB:DPE solution exhibited a finely dispersed surface topology with a much smoother surface (rms roughness values of 1.67 and 1.87 nm, respectively).

**Fig. 7:**
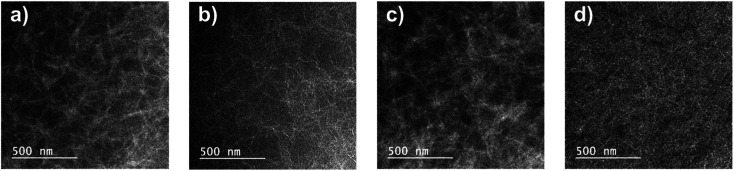
FE-TEM images of polymer:PC_71_BM blend films with DPE use: (a) PDPP2DT-F2T2, (b) PDPPF2T2DPP-T2, (c) PDPP-F2T2DPP-TVT, and (d) PDPPF2T2DPP-DTT.

To examine the morphology of polymer:PC_71_BM blend films processed with DPE additive, FE-TEM images were also taken as shown in Fig. 7. Considerable differences in the morphology were observed; nevertheless, all of the blend films had interconnected fibril structures, which were formed by DPP polymers.^17,28,33^ The PDPP2DT-F2T2:PC_71_BM and PDPPF2T2DPP-TVT:PC_71_BM films contained thick aggregated nanofibers (Fig. 7a and c). Due to the large polymer aggregation, the effective interfacial area between the polymer chains and PC_71_BM molecules for efficient exciton separation would have been relatively reduced, resulting in the moderate PCEs. The PDPPF2T2DPP-T2:PC_71_BM film had sparsely distributed very narrow nanofibers (Fig. 7b), which is consistent with the lowest carrier mobility and the lowest PCE in this study. In comparison with other blend films, the PDPPF2T2DPP-DTT:PC_71_BM blend film had well developed fibrillary structures with an even distribution (Fig. 7d). This type of nanofibrillar crystalline morphology is essential for achieving high device performance because it allows efficient charge transport. It is worth noting that the PDPPF2T2DPP-DTT polymer had fibrils and at the same time good miscibility with PC_71_BM domains. Considering that the DPP polymers in this study were not significantly different in terms of their geometries, it appears that a fused ring structure of the DTT moiety might facilitate π–π interactions between the PDPPF2T2DPP-DTT polymer chains and PC_71_BM molecules.

**Fig. 8:**
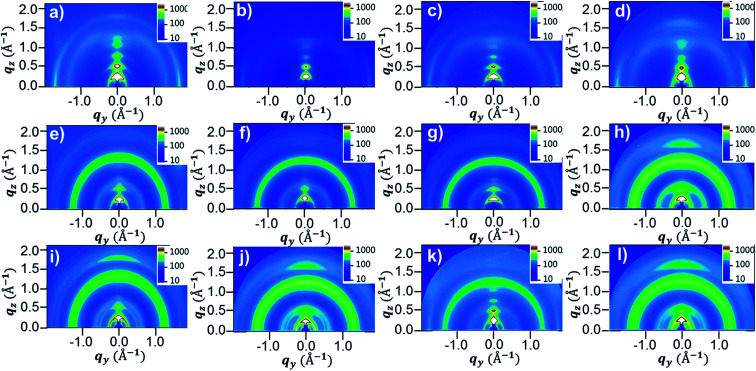
GIWAXS morphological data of (a–d) pristine polymer films, (e–h) polymer:PC_71_BM blend films without DPE use and (i–l) polymer:PC_71_BM blend films with DPE use. (a, e and i) PDPP2DT-F2T2, (b, f and j) PDPPF2T2DPP-T2, (c, g and k) PDPPF2T2DPP-TVT, and (d, h and l) PDPPF2T2DPP-DTT.

To further investigate the morphologies of the four DPP polymer-based blend films in terms of the orientation and packing of polymer chains, 2D-GIWAXS images were obtained. Fig. 8 and S15† show the 2D-GIWAXS images and in-plane and out-of-plane line-cut profiles of pristine polymers and polymer:PC_71_BM blend films without and with DPE addition. The extracted 2D-GIWAXS scattering features are summarized in Table S2.† The patterns of the pristine DPP polymer films were similar, showing strong lamellar scattering up to (500) in the out-of-plane direction with an edge-on orientation. From the out-of-plane (100) peaks, the estimated lamellar *d*-spacing values were similar (23.43, 21.95, 21.95, and 22.11 Å for PDPP2DT-F2T2, PDPPF2T2DPP-T2, PDPPF2T2DPP-TVT, and PDPPF2T2DPP-DTT, respectively), as expected based on the chemical structures. All of the pristine DPP polymer films also demonstrated strong (010) π–π stacking peaks in the in-plane direction with the same *d*-spacing distance (3.63 Å). In case of the polymer:PC_71_BM blend films without DPE addition, there was no large difference in the packing structure of the PDPP2DT-F2T2, PDPPF2T2DPP-T2, and PDPPF2T2DPP-TVT polymers; the lamellar and π–π stacking peaks were significantly decreased in the three DPP polymer:PC_71_BM blend films. In contrast, the PDPPF2T2DPP-DTT:PC_71_BM blend film exhibited clear π–π stacking in the out-of-plane direction. Upon the use of the DPE additive, we observed that π–π stacking peaks in the out-of-plane direction reappeared for the PDPP2DT-F2T2:PC_71_BM and PDPPF2T2DPP-T2:PC_71_BM blend films, and they became more pronounced for the PDPPF2T2DPP-DTT:PC_71_BM blend film. At the same time, (100) peaks in the in-plane direction were sharply increased for all of the DPP polymer:PC_71_BM blend films. These changes suggest that the DPE additive could facilitate the ordering and stacking of DPP polymer chains while promoting a face-on orientation. Because the face-on orientation is beneficial for charge transport and extraction in the vertical direction, these results clearly supported the high photovoltaic performance of the PDPP2DT-F2T2, PDPPF2T2DPP-T2, and PDPPF2T2DPP-DTT devices compared with that of the PDPPF2T2DPP-T2 device.

## 4. Conclusions

We investigated the photovoltaic properties of newly designed DPP-based D_1_–A–D_2_–A-type terpolymers (PDPPF2T2DPP-T2, PDPPF2T2DPP-TVT, and PDPPF2T2DPP-DTT; F2T2 as the D_1_ unit and either T2, TVT, or DTT as the D_2_ unit) as well as the D–A-type PDPP2DT-F2T2 polymer. Depending on the D_2_ unit, electronic levels and bandgaps were finely modulated; the terpolymers exhibited slightly higher lying HOMO energy levels. Measurement of photovoltaic properties revealed that the PSC containing the PDPPF2T2DPP-DTT:PC_71_BM blend film had the highest PCE (6.35%) with DPE addition. This good photovoltaic performance may be attributed to the face-on crystalline features of the PDPPF2T2DPP-DTT polymer in the polymer:PC_71_BM blend film, which could facilitate good carrier transport, and the close interaction of the long nanoscale fibrils of the PDPPF2T2DPP-DTT polymer with the PC_71_BM domains. We emphasize that the PCE of the PDPPF2T2DPP-DTT device was much higher than that of the D–A-type PDPP2DT-F2T2 device (4.78%). As the DTT unit is a stronger donor than the F2T2 unit, the *V*_oc_ value of the PDPPF2T2DPP-DTT device was slightly lower. However, the DTT unit generated a more ideal morphology and chain orientation; thus, *J*_sc_ values were markedly improved from 8.29 to 14.14 mA cm^−2^. This study highlights the great potential of using D_1_–A–D_2_–A-type conjugated polymers rather than simple D–A-type polymers to tailor electronic properties and morphology for fabricating high-performance PSCs.

## Conflicts of interest

There are no conflicts to declare.

## Supplementary Material

RA-009-C9RA08858J-s001
