# Supplementary material for: Regio-regular alternating diketopyrrolopyrrole-based D1–A–D2–A terpolymers for the enhanced performance of polymer solar cells
Source: RSC Adv. 2019 Dec 18;9(72):42096–109. doi: 10.1039/c9ra08858j (PMC9076513; doi:10.1039/c9ra08858j)
Supplement: RA-009-C9RA08858J-s001 [file RA-009-C9RA08858J-s001.pdf]

Electronic Supplementary Material (ESI) for RSC Advances.
This journal is © The Royal Society of Chemistry 2019

ELECTRONIC SUPPLEMENTARY INFORMATION

Regio-regular Alternating Diketopyrrolopyrrole-based D_1-A-D_2-A Terpolymers for the Enhanced Performance of Polymer Solar Cells

Myeongjae Lee,[‡a] Taehyo Kim,[‡b] Hoai Van T. Nguyen,[c] Hye Won Cho,[b] Kyung-Koo Lee,[c] Jong-Ho Choi,[a] BongSoo Kim,[*d] and Jin Young Kim[*b]

[a]Department of Chemistry, Korea University, 145 Anam-ro, Seongbuk-gu, Seoul 02841, Republic of Korea

[b]School of Energy and Chemical Engineering, Ulsan National Institute of Science and Technology (UNIST), 50 UNIST-gil, Ulsan 44919, Republic of Korea

[c]Department of Chemistry, Kunsan National University, 558 Daehak-ro, Gunsan-si, Jeollabuk-do 54150, Republic of Korea

[d]Department of Chemistry, Ulsan National Institute of Science and Technology (UNIST), 50 UNIST-gil, Ulsan 44919, Republic of Korea

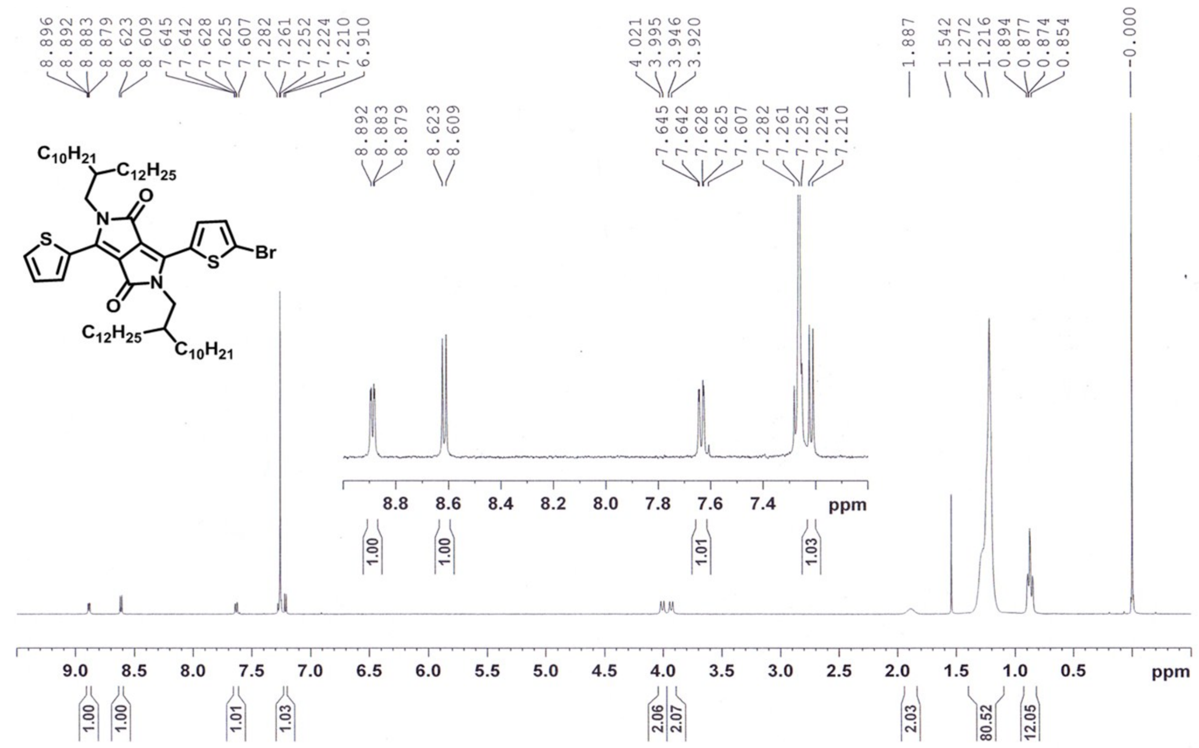

Figure S1. ^{1}H-NMR spectrum of DPP2DT-Br

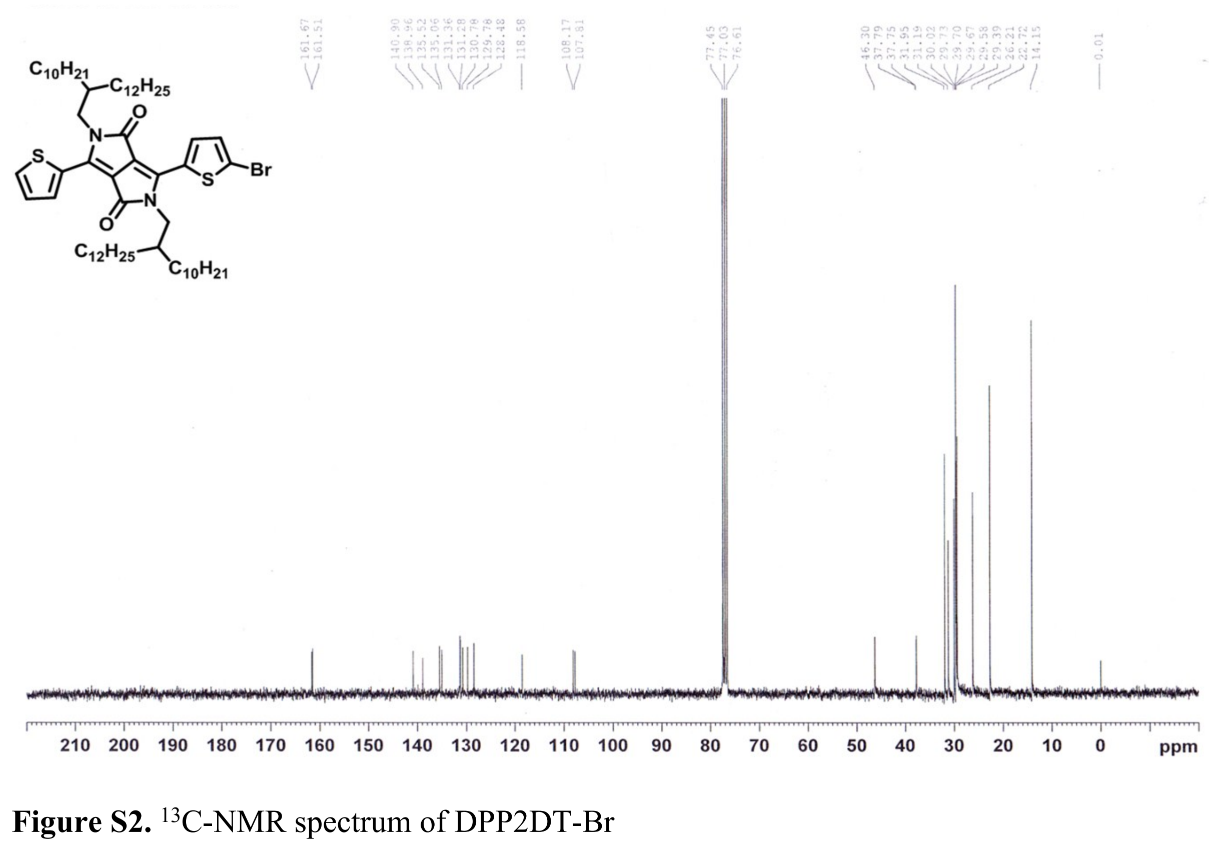

Figure S2. ^{13}C-NMR spectrum of DPP2DT-Br

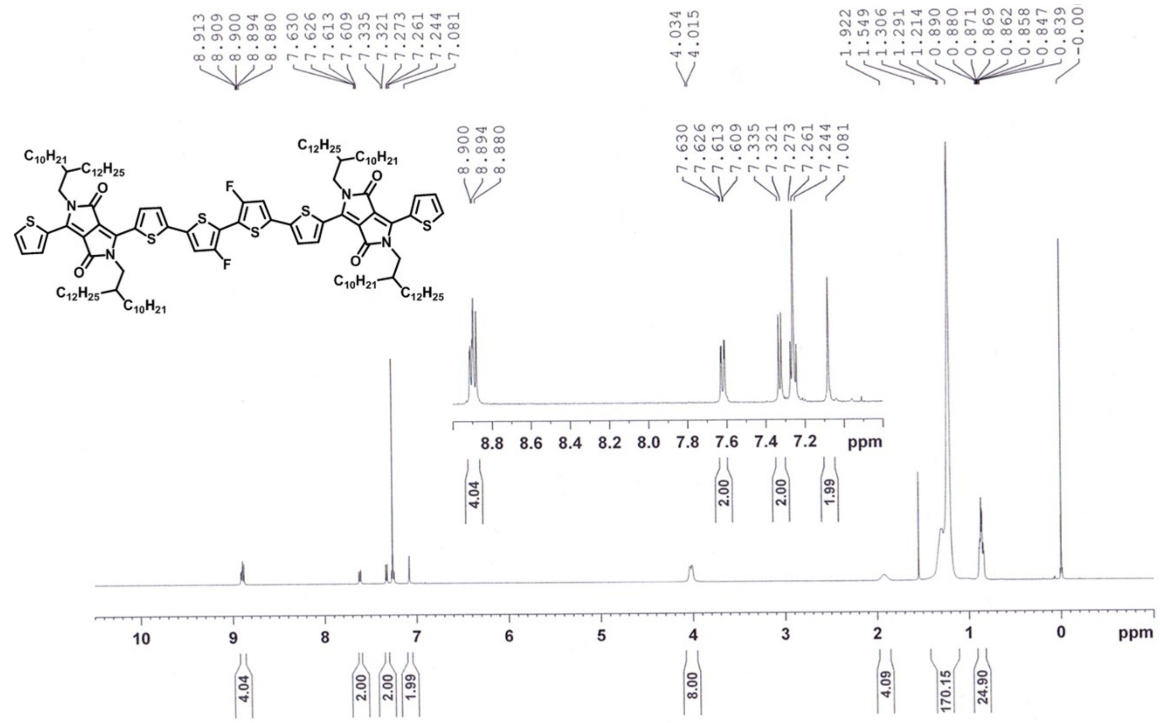

Figure S3. ^{1}H-NMR spectrum of DPPF2T2DPP

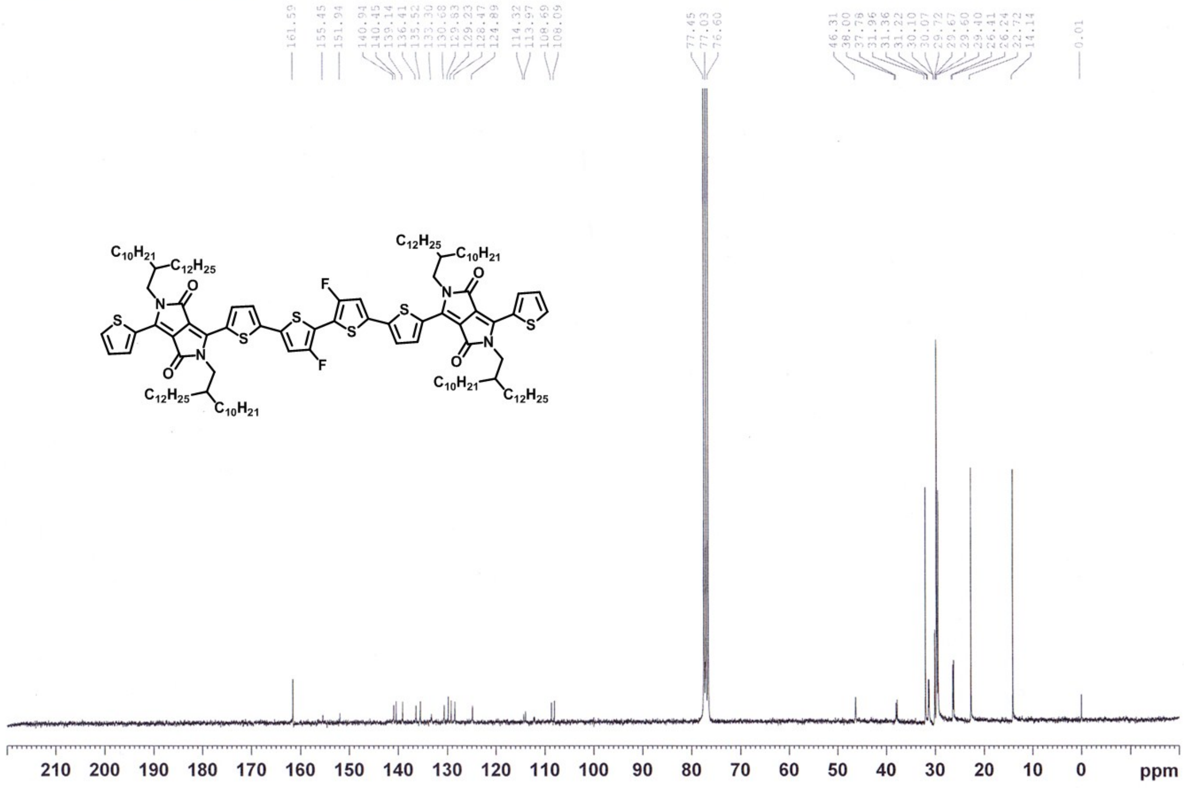

Figure S4. ^{13}C-NMR spectrum of DPPF2T2DPP

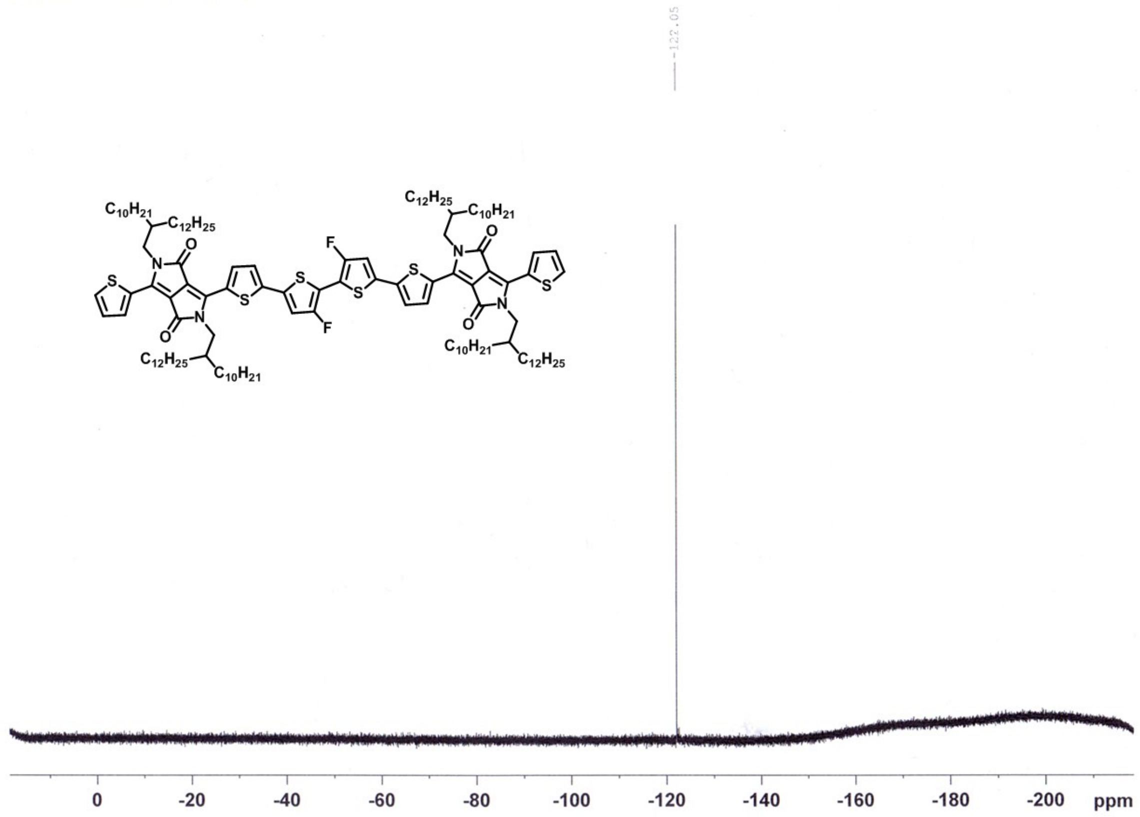

Figure S5. ^{19}F-NMR spectrum of DPPF2T2DPP

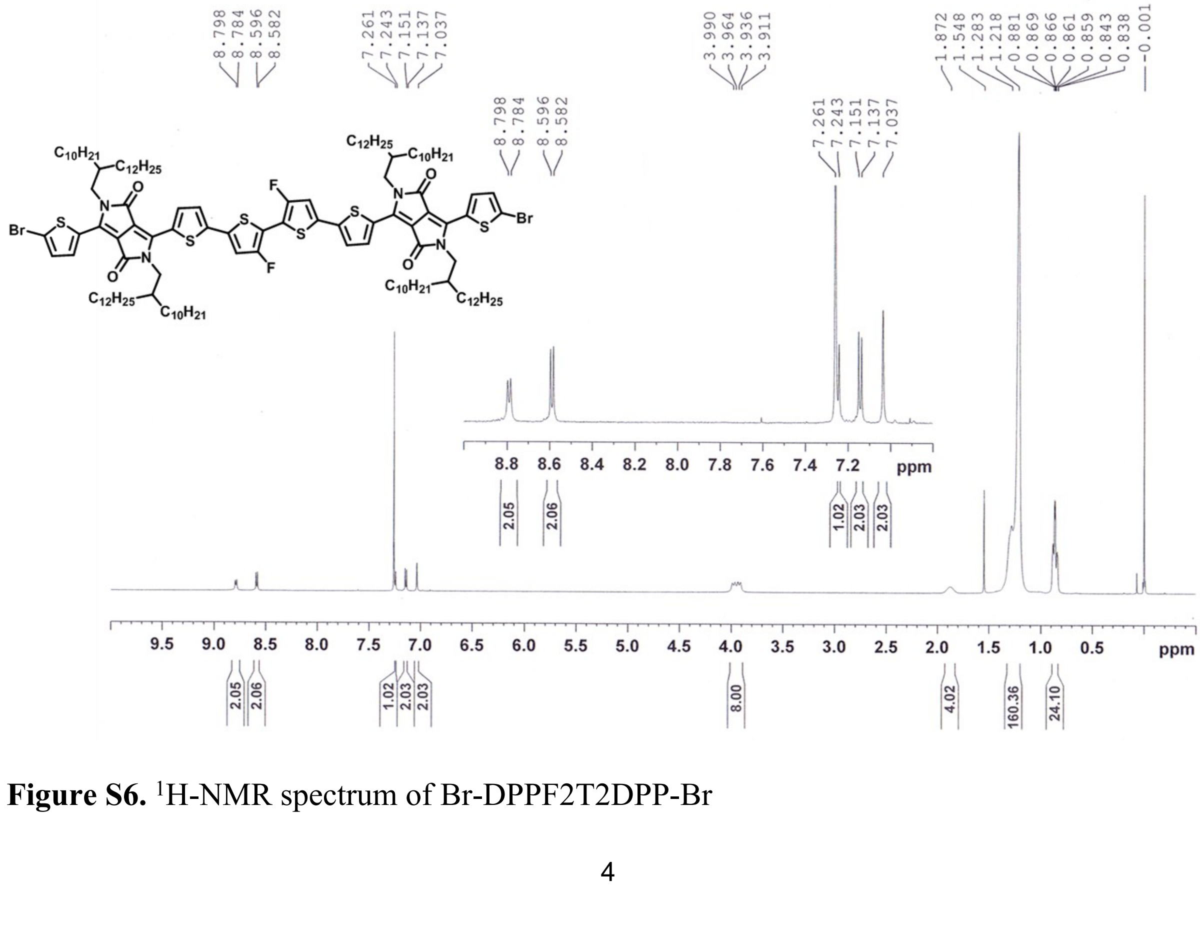

Figure S6. ^{1}H-NMR spectrum of Br-DPPF2T2DPP-Br

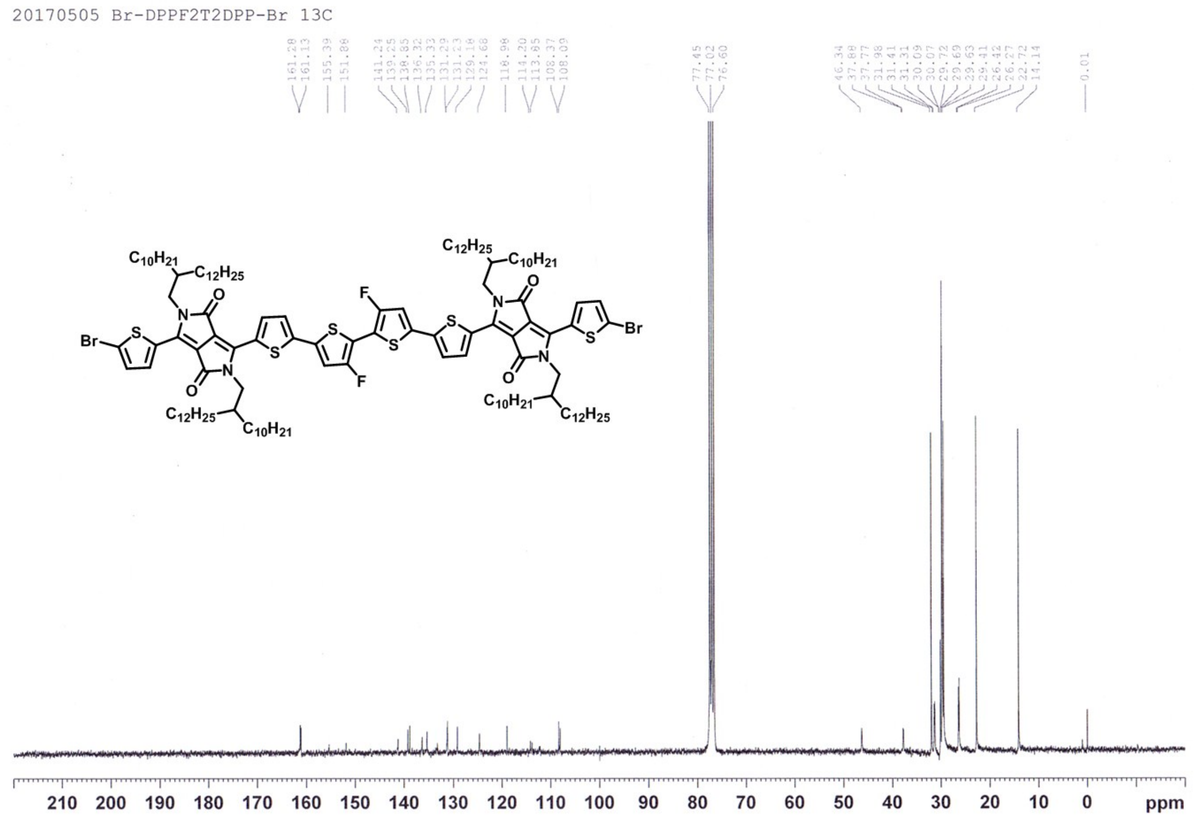

Figure S7. ^{13}C-NMR spectrum of Br-DPPF2T2DPP-Br

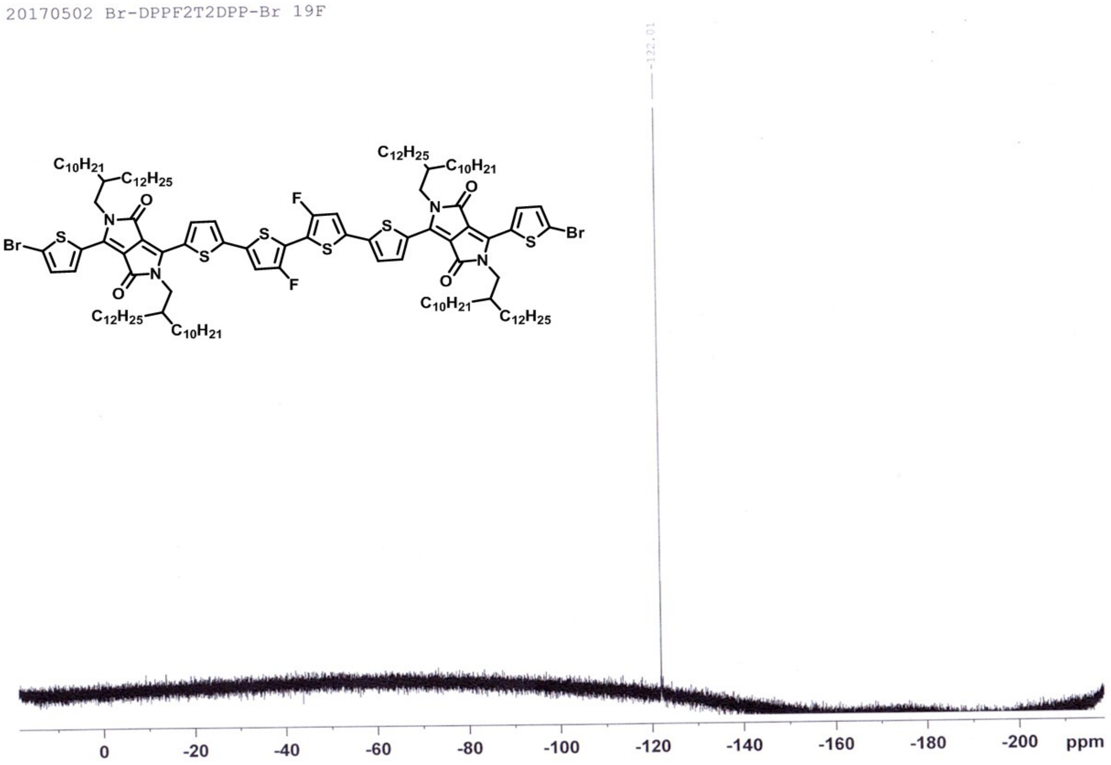

Figure S8. ^{19}F-NMR spectrum of Br-DPPF2T2DPP-Br

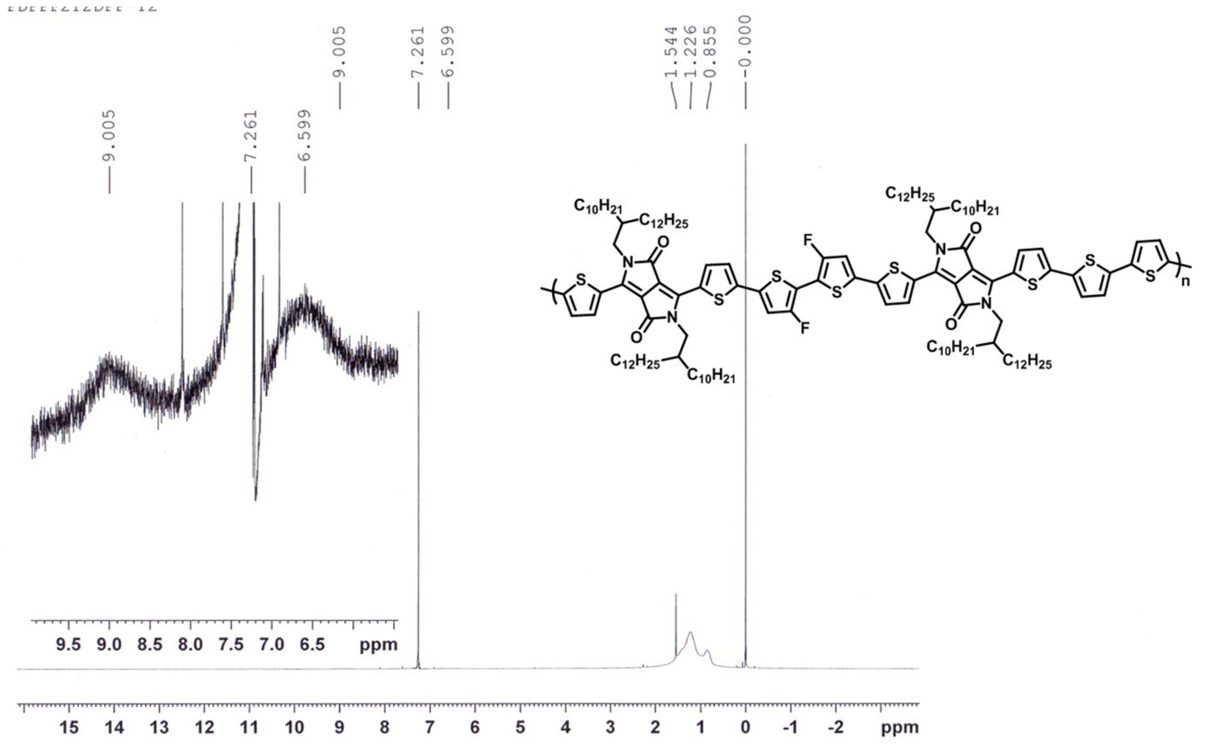

Figure S9. ^{1}H-NMR spectrum of PDPPF2T2DPP-T2

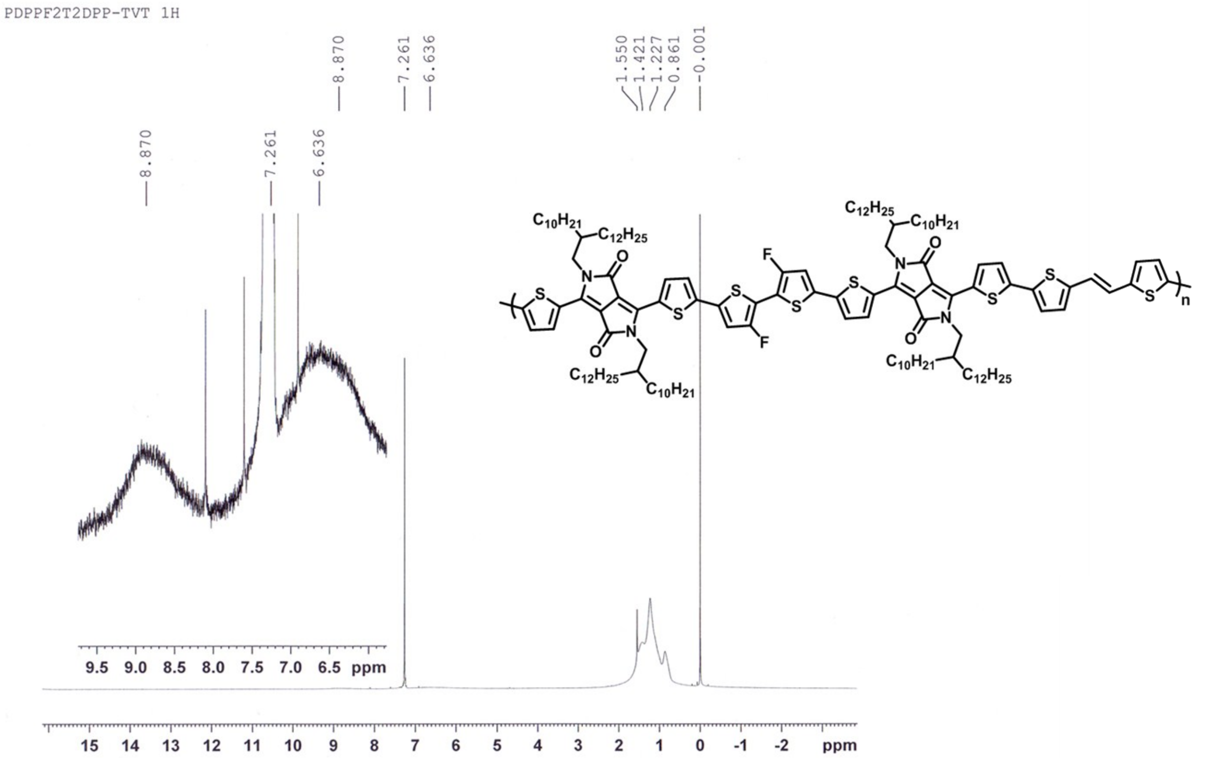

Figure S10. ^{1}H-NMR spectrum of PDPPF2T2DPP-TVT

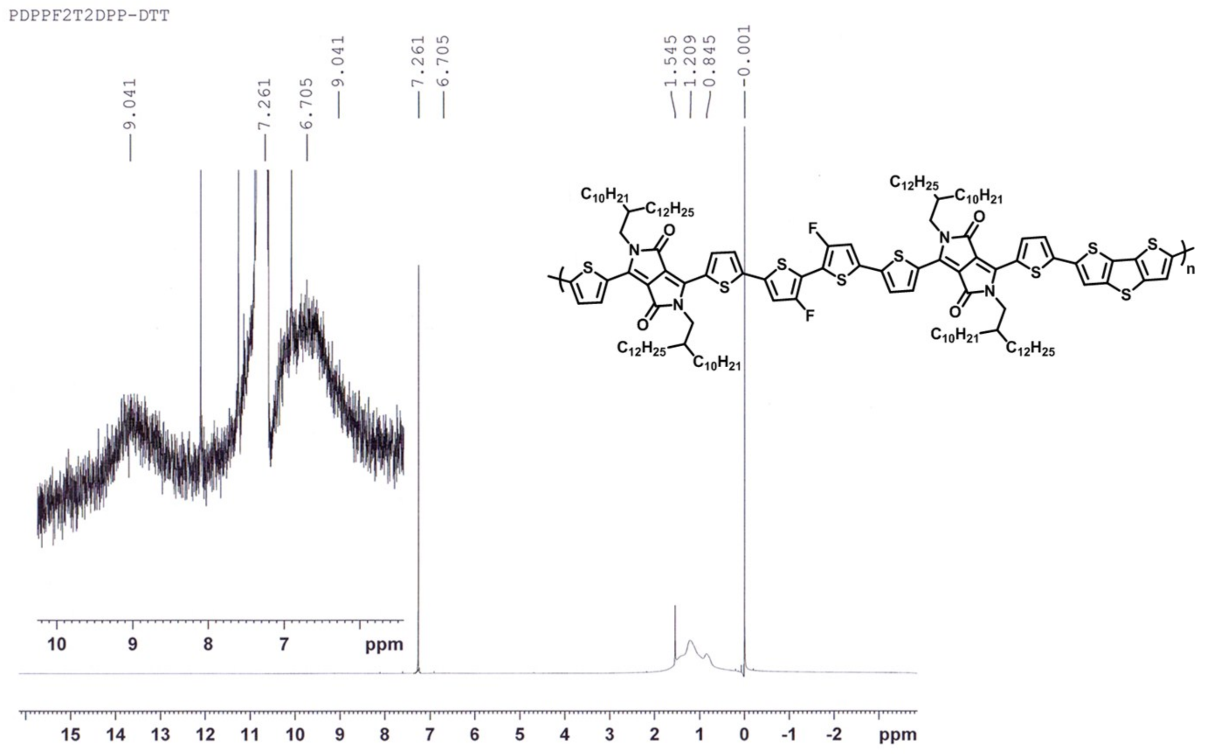

Figure S11. ^{1}H-NMR spectrum of PDPPF2T2DPP-DTT

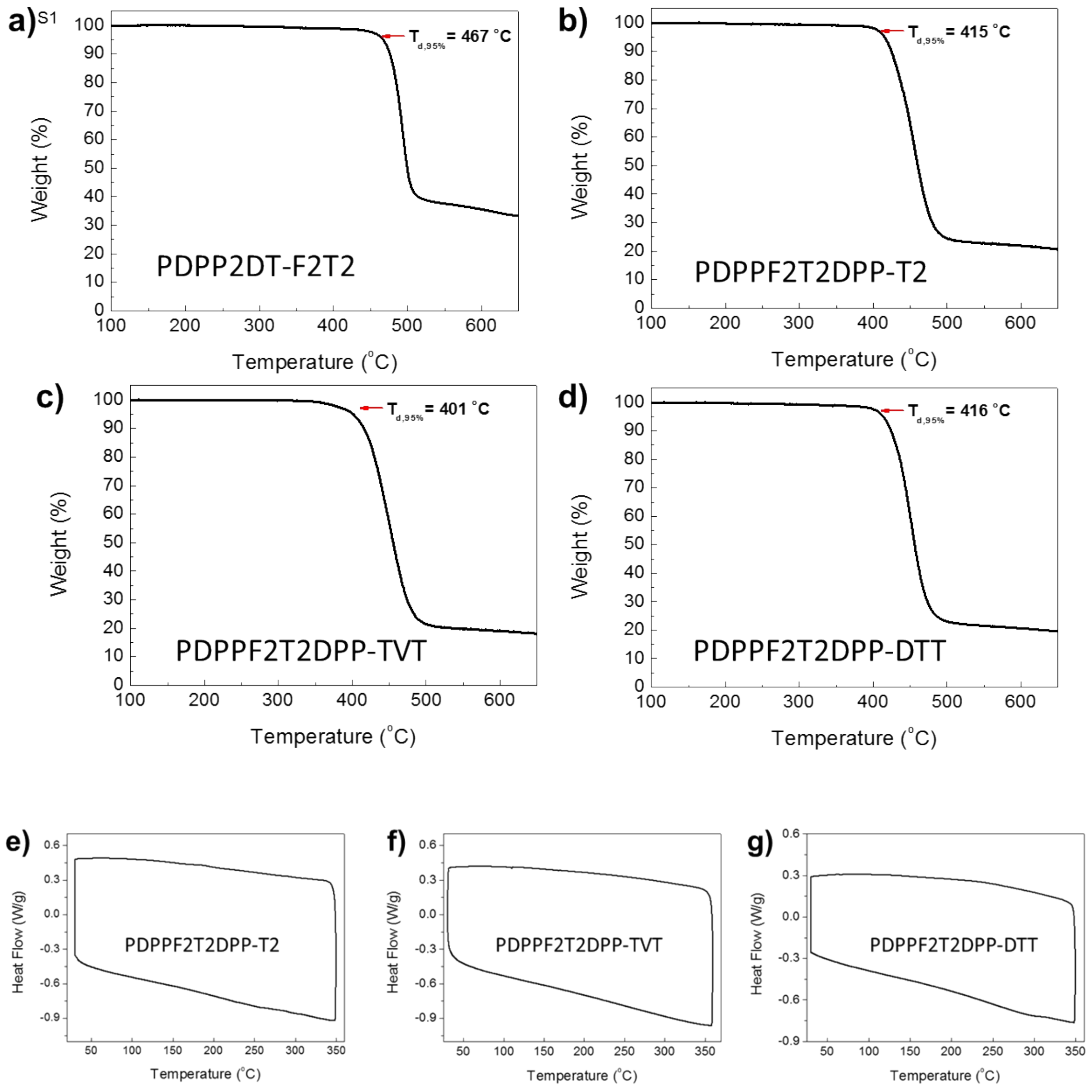

Figure S12. (a-d) TGA and (e-g) DSC curves of the DPP-polymers in this study.

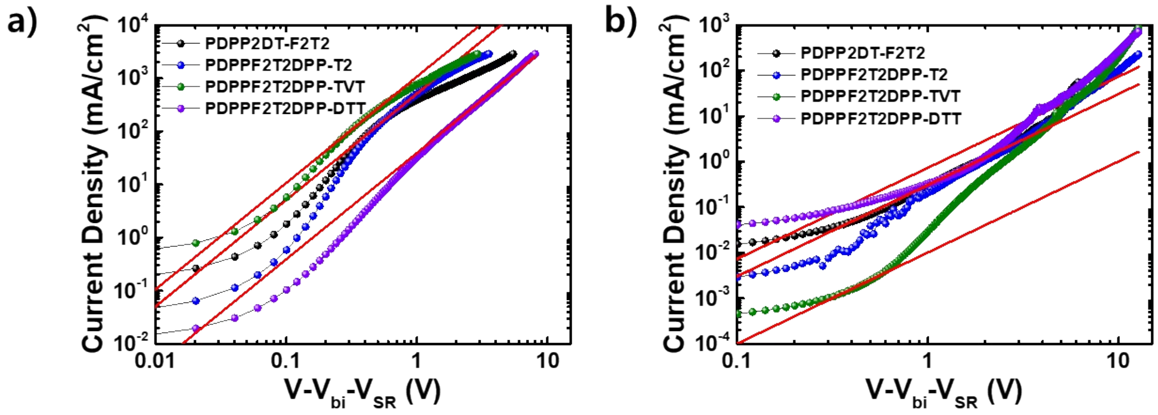

Figure S13. Measured *J*–*V* characteristics with red lines fitted by SCLC model for (a) hole-only devices and (b) electron-only devices containing the pristine polymer films.

Table S1 Hole and electron mobilities of polymer films, estimated using the SCLC method

Polymer	μ_h [$cm^2s^{-1}V^{-1}$]	μ_e [$cm^2s^{-1}V^{-1}$]
PDPP2DT-F2T2	1.56×10^{-3}	4.39×10^{-8}
PDPPF2T2DPP-T2	1.69×10^{-3}	1.35×10^{-7}
PDPPF2T2DPP-TVT	1.18×10^{-3}	3.10×10^{-9}
PDPPF2T2DPP-DTT	2.46×10^{-3}	7.00×10^{-7}

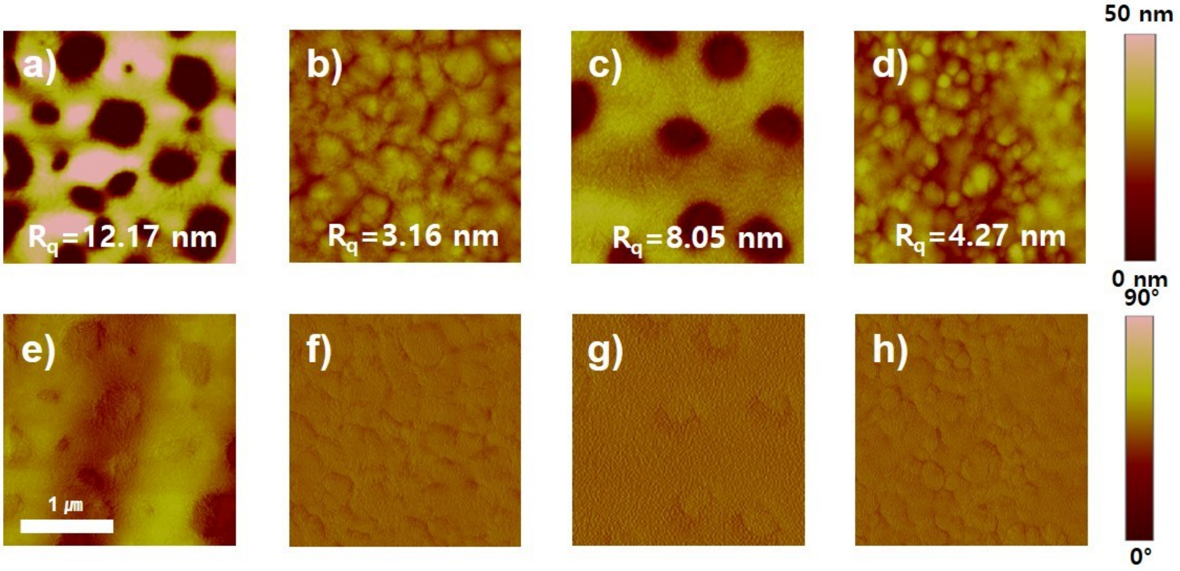

Figure S14. Surface morphology of polymer:$PC_{71}BM$ films without DPE additives. AFM (a–d) topographic and (e–h) phase images: (a,e) PDPP2DT-F2T2, (b,f) PDPPF2T2DPP-T2, (c,g) PDPPF2T2DPP-TVT, and (d,h) PDPPF2T2DPP-DTT.

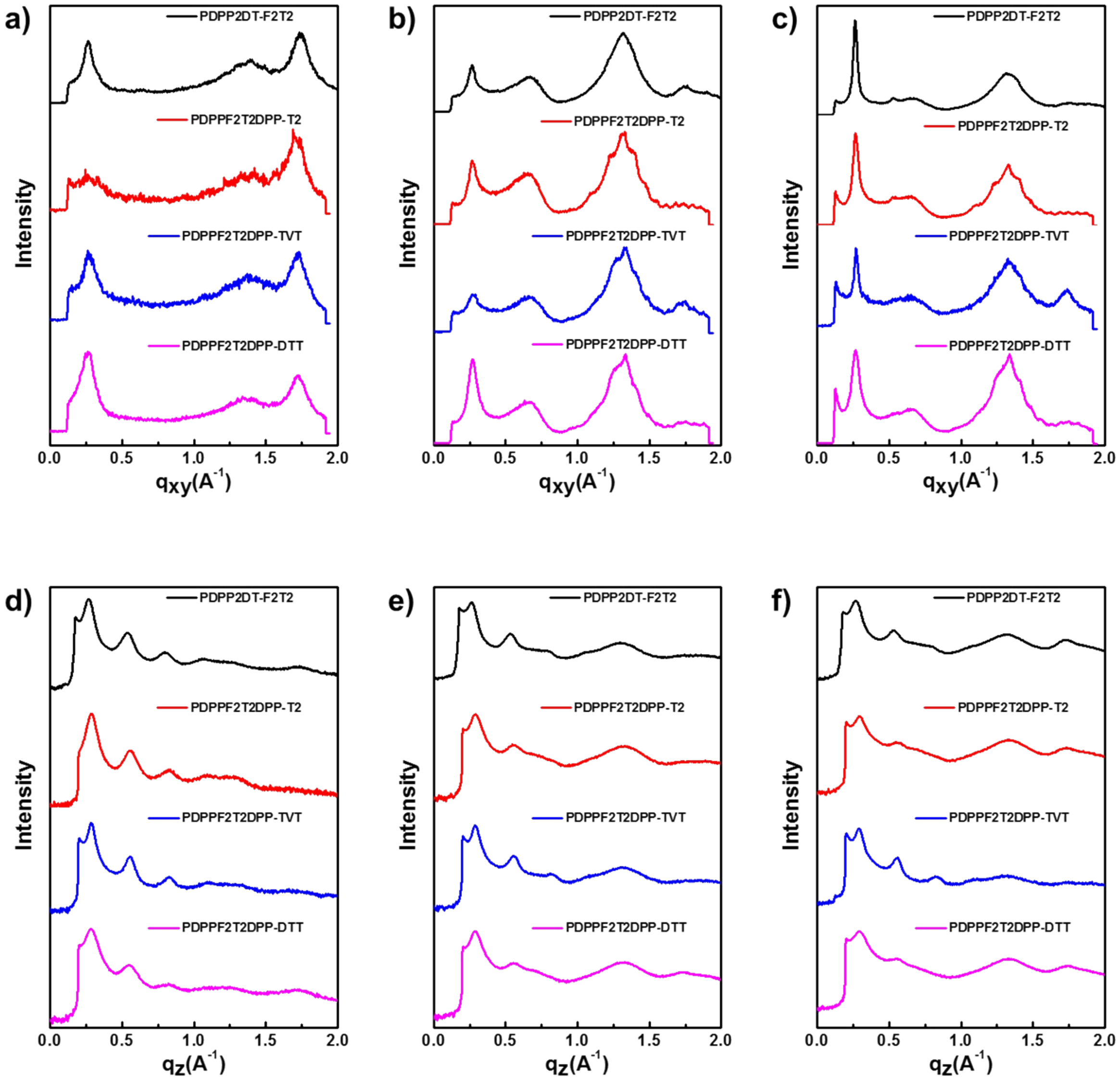

Figure S15. GIWAXS line-cut profiles of (a,d) pristine polymer films, (b,e) polymer:$PC_{71}BM$ blend films without DPE use, and (c,f) polymer:$PC_{71}BM$ blend films with DPE use; (a-c) in-plane profiles and (d-f) out-of-plane profiles)

Table S2. Packing parameters derived from GIWAXS patterns.

	Polymer	Plane	Crystallographic parameters			
			Lamellar spacing		π-π stack	
			q (Å^{-1})	d-spacing (Å)	q (Å^{-1})	d-spacing (Å)
Pristine polymer	PDPP2DT-F2T2	In	0.262	23.96	1.73	3.63
		Out	0.268	23.43	1.72	3.65
	PDPPF2T2DPP-T2	In	0.275	22.83	1.73	3.63
		Out	0.286	21.95	-	-
	PDPPF2T2DPP-TVT	In	0.263	23.87	1.73	3.63
		Out	0.286	21.95	1.71	3.67
	PDPPF2T2DPP-DTT	In	0.263	23.87	1.73	3.63
		Out	0.284	22.11	1.73	3.63
Polymer:$PC_{71}BM$ Blend	PDPP2DT-F2T2	In	0.264	23.78	1.74	3.60
		Out	0.264	23.78	-	-
	PDPPF2T2DPP-T2	In	0.267	23.52	-	-
		Out	0.290	21.65	-	-
	PDPPF2T2DPP-TVT	In	0.275	22.83	1.75	3.58
		Out	0.290	21.65	-	-
	PDPPF2T2DPP-DTT	In	0.273	23.00	-	-
		Out	0.286	21.95	1.72	3.65
Polymer:$PC_{71}BM$ Blend with DPE	PDPP2DT-F2T2	In	0.262	23.96	1.70	3.69
		Out	0.268	23.43	1.72	3.65
	PDPPF2T2DPP-T2	In	0.265	23.69	-	-
		Out	0.296	21.21	1.75	3.58
	PDPPF2T2DPP-TVT	In	0.269	23.34	1.73	3.63
		Out	0.290	21.65	-	-
	PDPPF2T2DPP-DTT	In	0.267	23.52	-	-
		Out	0.296	21.21	1.75	3.58
